# Role of FOXC1 in regulating APSCs self-renewal via STI-1/PrP^C^ signaling

**DOI:** 10.7150/thno.35619

**Published:** 2019-08-15

**Authors:** Yi-Hui Lee, Hsu-Tung Lee, Chien-Lin Chen, Chi-Hao Chang, Chung Y. Hsu, Woei-Cherng Shyu

**Affiliations:** 1 Translational Medicine Research Center, Drug Development Center and Department of Neurology, China Medical University & Hospital, Taichung, Taiwan; 2 Department of Neurosurgery, Taichung Veterans General Hospital, Taichung, Taiwan; 3 Graduate Institute of Medical Sciences, National Defense Medical Center, Taipei, Taiwan; 4 Graduate Institute of Biomedical Science and Drug Development Center, China Medical University, Taichung, Taiwan; 5 Department of Occupational Therapy, Asia University, Taichung, Taiwan

**Keywords:** forkhead box family C1 (FOXC1), stress-inducible protein 1 (STI-1), arachnoid-pia stem cells (APSCs)

## Abstract

Forkhead box protein C1 (FOXC1) is known to regulate developmental processes in the skull and brain.

**Methods**: The unique multipotent arachnoid-pia stem cells (APSCs) isolated from human and mouse arachnoid-pia membranes of meninges were grown as 3D spheres and displayed a capacity for self-renewal. Additionally, APSCs also expressed the surface antigens as mesenchymal stem cells. By applying the FOXC1 knockout mice and mouse brain explants, signaling cascade of FOXC1-STI-1-PrP^C^ was investigated to demonstrate the molecular regulatory pathway for APSCs self-renewal. Moreover, APSCs implantation in stroke model was also verified whether neurogenic property of APSCs could repair the ischemic insult of the stroke brain.

**Results**: Activated FOXC1 regulated the proliferation of APSCs in a cell cycle-dependent manner, whereas FOXC1-mediated APSCs self-renewal was abolished in FOXC1 knockout mice (*FOXC1^-/-^* mice). Moreover, upregulation of STI-1 regulated by FOXC1 enhanced cell survival and self-renewal of APSCs through autocrine signaling of cellular prion protein (PrP^C^). Mouse brain explants STI-1 rescues the cortical phenotype *in vitro* and induces neurogenesis in the *FOXC1*^-/-^ mouse brain. Furthermore, administration of APSCs in ischemic brain restored the neuroglial microenvironment and improved neurological dysfunction.

**Conclusion**: We identified a novel role for FOXC1 in the direct regulation of the STI-1-PrP^C^ signaling pathway to promote cell proliferation and self-renewal of APSCs.

## Introduction

The leptomeningeal cells (arachnoid-pia cells), which cover the entire CNS, could indirectly regulate neural progenitor cell (NPC) proliferation to stimulate cortical neurogenesis 1, 2 and modulate cell migration and differentiation in multiple regions of the embryonic and infant brain and skull 3 through secretion of soluble factors 4. Importantly, adult NPCs located in the leptomeninges or neocortical layer 1 could be isolated from these nonconventional neurogenic areas under ischemia or injury conditions 5-9. With regard to corticogenesis and osteogenesis, previous studies have demonstrated that meningeal cells, especially the forebrain meningeal cells that originated from pluripotent neural crest stem cells (NCSCs), contributed to the development and growth of embryonic progenitor cells in the hippocampal dentate gyrus 10, 11. To investigate the regulatory mechanisms and multipotent potential under steady state, it is important to study the characteristics of the stem cells derived from arachnoid-pia tissue (arachnoid-pia stem cells-APSCs).

*FOXC1*, a single exon gene encoding a 553 amino acid protein 12 is a member of the forkhead box (FOX) family of transcription factors 13. FOXC1 plays an important role in regulating developmental processes including somatic, cardiovascular, renal, ocular and cerebellar development 14-16. In a previous study, mice with *FOXC1* gene mutations showed meningeal layer abnormalities with severe brain and skull defects. Thus, FOXC1 plays a significant role in meninges-based structural development (arachnnoid-pia cells) and further regulates embryogenesis of the skull and cerebral cortex 17, 18. A report further found that loss of meningeal-derived retinoic acid in FOXC1 null mice impaired normal neural progenitor cell proliferation and differentiation thus disturbing corticogenesis 1. Since the above evidence only notes the relationship between FOXC1 and arachnoid-pia cells with no molecular interpretation, we proposed to investigate the regulatory mechanisms of FOXC1 in APSC self-renewal and proliferation.

The migration/proliferation of cerebellar precursor cells of the external germinal layer (EGL) are affected by stromal cell-derived factor 1 (SDF-1α) secreted from the arachnoid-pia cells of the meninges 4. In our previous study, we demonstrated that strong interactions between CXCR4 and cellular prion protein (PrP^C^) with SDF-1α upregulation in the olfactory ensheathing cell-implanted stroke brain triggered neuroplastic signals in response to hypoxia and ischemia 19. Regarding the ligand of PrP^C^, stress-inducible protein 1 (STI-1) exhibited autocrine/paracrine activity that induced neurotrophic effects 20-23 against cell death 24. Importantly, it is evident that expression of PrP^C^ is found in leptomeninges (PLoS Pathogens 2012;6: e1000800), and the STI-1/PrP^C^ signaling complex is essential for the self-renewal of neural progenitor cells (NPCs) by regulating their proliferation and stemness capacity 20. In this study, we hypothesized that FOXC1 plays a significant role in the self-renewal of APSCs and contributes to embryonic and adult neurogenesis. We further validate whether STI-1 is a target of FOXC1 to stimulate PrP^C^-mediated APSC proliferation and self-renewal.

## Materials and Methods

### Primary cultures of sphere-like arachnoid-pia stem cells (APSCs)

Adult human arachnoid-pia membrane from neurosurgical specimens were separated from the dura meninges (5 mm^3^, 0.5 gm in weight) and collected in sterile boxes containing Hanks' balanced salt solution (HBSS; Gibco/BRL) for primary culture within 24 hours. Protocols for sampling adult human meninges were approved by the Institutional Review Board of China Medical University and Hospital, Taichung, Taiwan. Written informed consent was obtained from all patients. In brief, the tissue was carefully dissected into small pieces under a dissecting microscope and placed in a phosphate-buffered solution at room temperature. The tissue was then ground with a dissection scalpel and transferred into 10 ml Dulbecco's Modified Eagle Medium (DMEM)/F12 medium containing trypsin and EDTA and shaken at 37°C in a water bath for 5 minutes. It was then rinsed with DMEM/F12 solution and triturated with a fire-polished Pasteur pipette. The ground tissue explants were collected by centrifugation at 600 *g* for 10 minutes. In adherent culture, the resulting pellet was resuspended in DMEM/F12 medium (Gibco), 10% heat-inactivated fetal calf serum (FCS) (Gibco) and 1% penicillin/streptomycin (100 U/mL) at 300,000 cells per ml of culture medium. The tissue explant was placed in a 75 cm^2^ flat flask and incubated in 5% CO_2_ at 37°C. The tissue was left undisturbed for 5-7 days to allow for migration of the cells from the explants and subsequently regarded as “human arachnoid-pia stem cells” (APSCs). After 10 days of adherent culture, clear colony-forming units could be detected. In sphere cultures, tissue explants were seeded in 3 mL of neurosphere culture medium with Neurobasal medium containing B27 medium supplement (Gibco), 1% N2 supplement (Gibco), 10 ng/mL FGF-2 (R&D Systems), 10 ng/mL EGF (R&D Systems) and 1% penicillin/streptomycin (100 U/mL). These primary sphere-forming arachnoid-pia cells called APSs were passaged once a week for three to four weeks.

In addition, arachnoid-pia membrane samples from heterozygous mice (*FOXC1^+/-^*) or homozygous mice (*FOXC1^-/-^*) of the null mutation *FOXC1^lacZ^* 17 and their normal littermates (NLs) (2 mm^3^, 0.1 gm in weight) were prepared for isolation using explant culture. All isolation procedures were performed according to a protocol approved by the Institutional Animal Care Committee at the China Medical University. In brief, the arachnoid-pia tissue of the meninges of the frontal region was isolated aseptically from eight-week adult mice (Am) or mice fetuses at 12-13 embryonic days. The tissue was washed in PBS and plated in tissue culture dishes with media as mentioned above (10% FBS, 1% L-glutamine, 0.5% gentamycin in DMEM/F12) to allow outgrowth of sphere-forming cells (AmAPSCs or mAPSCs) from these explants and to gradually induce formation of mouse arachnoid-pia spheres (AmAPs or mAPSs). Cells from either NLs or *FOXC1^-/-^* mice were maintained at subconfluent levels and cultured at 37^o^C with 5% CO_2_. Only passage 5 (p5) or less were used for these experiments.

### Immunocytochemistry, alkaline phosphatase staining and flow cytometric analysis

For immunocytochemistry, cell cultures from APSCs and mAPSCs were washed with PBS and fixed for 30 minutes at room temperature in 1% paraformaldehyde. After washing with PBS, the fixed cells were treated for 30 minutes with blocking solution (10 g/L BSA, 0.03% Triton X-100, and 4% serum in PBS). Cells were incubated overnight at 4°C with an antibody against FOXC1 (1:200, Novus Biologicals), Wnt1 (1:300, R&D Systems), OCT4 (1:300, Abcam), SOX2 (1:100, R&S System), SSEA4 (1: 200, Milipore), SSEA3 (1:200, Millipore), Nanog (1:200, Millipore), nestin (1:300, Millipore), NGFRp75 (1:100, Millipore), Musashi-1 (1:100, Millipore), GFAP (1:400, Sigma), Tuj-1 (1:200, Chemicon), MAP-2 (1:300, Chemicon), O4 (1:300, Millipore), Ki67 (1:300, Chemicon), STI-1 (1:200, Santa Cruz Biotechnology), or PrP^C^ (1:200, Santa Cruz Biotechnology) conjugated with FITC or Cy-3 (1:500, Jackson ImmunoResearch). Finally, several of the preparations were lightly counterstained with DAPI and then mounted. The preparations were analyzed with a Carl Zeiss LSM510 laser-scanning confocal microscope. In addition, alkaline phosphatase (ALP) staining was performed using the Leukocyte Alkaline Phosphatase kit (Sigma) according to the manufacturer's instructions.

For flow cytometry, APSs were dissociated by incubation in 0.25% trypsin-EDTA, 1 mg/ml collagenase type 1a (Sigma, USA), 1 mg/ml collagenase IV and 2% hyaluronidase (Sigma) for 5 minutes at 37°C. The cells were detached with 2 mM EDTA in PBS, washed with PBS containing 2% BSA and 0.1% sodium azide (Sigma), and incubated with the appropriate antibody conjugated with fluorescein isothiocyanate (FITC) or phycoerythrin (PE) including CD13, CD29, CD34, CD44, CD45, CD73, CD90, CD105, CD117, CD166, HLA-ABC, HLA-DR (BD Bioscience), NGFRp75 (Millipore), OCT4 (Abcam), SSEA1 (Milipore), Nanog (Millipore), SSEA4 (Milipore), SSEA3 (Millipore), SOX2 (R&S System), Tra-1-60 (BD Bioscience), and Tra-1-81 (Chemicon). The cells were analyzed using a FACSTAR^+^ flow cytometer (Becton Dickinson).

### Clonal sphere-forming assay

Clonal analysis was performed as previously described 25, 26. Mouse neural stem cells (mNSCs) that formed neurospheres served as positive controls. Spheres from APSs or mAPSs grown at high density were dissociated into a single cell suspension by incubation in 0.25% trypsin-EDTA, 1 mg/ml collagenase type 1a (Sigma, USA), 1 mg/ml collagenase IV and 2% hyaluronidase (Sigma) for 5 minutes at 37°C and reseeded into 96-well plates at 100 cells per ml of media. The density has been experimentally determined to produce a single clonal sphere that is equivalent to a single cell per well. Clonally derived spheres with the same numbers of seeding cells were counted and their diameters measured using bright-field illumination and image analysis software (MCID, Imaging Research). A minimum diameter cutoff of 40 μm was used in defining a sphere.

### Bromodeoxyuridine (BrdU) proliferation assay

For* in vitro* studies, cell proliferation was evaluated by measuring incorporation of BrdU using BrdU chemiluminescence ELISA kits (Roche) and further confirmed by standard trypan blue cell counting. After a 4- to 6-h starvation (medium without supplements), mAPSCs were returned to complete medium after starvation (another positive control) for 2 days and pulse loaded with 10 μM BrdU (Sigma-Aldrich) for 2 hours. NPCs were then incubated with anti-BrdU-peroxidase for 90 min and further developed with substrate solution for 3 min. The plates were read with a Lmax microplate luminometer (Molecular Devices). After subtracting the value of the blank (without BrdU loading), the results were analyzed and presented as percent (%) increase *vs*. positive control. For *in vivo* labeling, two different protocols of BrdU administration (pulse labeling and cumulative labeling) with 50 mg/kg BrdU intraperitoneally have been described previously 27. For BrdU immunohistochemistry, the brain section was pretreated with 2 N HCl and neutralized in 0.1 M boric acid, pH 8.5. After washing, sections were incubated with primary antibody for BrdU (1:50, BD Biosciences) overnight and then with the secondary antibody (1:200, Jackson ImmunoResearch Laboratories) for 1 h. After washing in PBS, pH 7.4, sections were mounted and analyzed by confocal microscopy (BX60; Olympus). Cells were scored as BrdU^+^ only when strong immunoreactivity was clearly detected specifically in the nucleus. The percentage of proliferating cells was determined by dividing the number of BrdU^+^ cells by the number of total nuclei. Additionally, the cell proliferation ability measured by Ki67 immunoreactivity (0.3 mg/ml, Ab15580, Abcam) was determined as the percentage of Ki67^+^ cells relative to all nuclei.

For *in vivo* cell cycle length studies, mice (E15) were injected with BrdU (50 mg/kg, i.p.), and brains were withdrawn 30 min later. Ki67^+^ cells were identified randomly in the SVZ on the basis of immunolabeling; subsequently, BrdU immunoreactive cells that colocalized with Ki67^+^ cells were identified 28. SVZ precursor cells were identified as those lying distant from the characteristic dense layer of BrdU^+^ precursors in the upper VZ as described 29. To study cell cycle exit and re-entry, mice were administered BrdU (50 mg/kg, i.p) and brains were recovered 24 hours later. The ratio of Ki67^+^BrdU^+^ double-labeled cells divided by the total number of BrdU^+^ cells was determined as previously described 30, 31.

### Self-renewal assays

To evaluate the self-renewal capacity of the mAPSCs, clonal colony forming assays measured the proportion of cells that were able to make new spheres. Single cells resuspended from spheres were plated into 96-well culture plates with 15 cells per well, and the number of newly formed spheres was counted after 7 days. We quantified the self-renewal potential by counting the number of secondary spheres generated from each primary neurosphere. In addition, secondary sphere formation capacity was measured by dissociating the primary spheres to single cells and by plating them into a 96-well plate. An average number of 4 separate spheres was counted in each experiment.

### TUNEL assay

Cellular apoptosis *in vitro* and *in vivo* were assayed using a commercial TUNEL (terminal deoxynucleotidyl transferase-mediated digoxigenin-dUTP nick-end labeling) staining kit (DeadEnd Fluorimetric TUNEL system; Promega) as previously described 32. We quantified the TUNEL data as the ratio of TUNEL^+^ cells per total nuclei.

### Cell-cycle analysis

Cells were fixed in 4% paraformaldehyde, permeabilized with 0.3% Triton X-100 and incubated with a propidium iodide staining (PI) solution (1.8 mg/ml RNase A, 50 μg/ml propidium iodide) for 3 hours to label DNA. Using a FACScan (BD Biosciences) flow cytometer, 10000 cells were sorted based on DNA content. The data were processed and the percentage of cells in each phase of the cell cycle were quantified using Cellquest Software (BD Biosciences) 33.

### Analysis of telomerase activity

Cell samples of mNSCs, APSCs and mAPSCs were prepared at 100 cells/cm^2^ in a 10-cm diameter dish for 5 days in order to quantitatively examine telomerase activity using a TeloTAGGG PCR ELISA kit (Roche) according to the manufacturer's protocol as previously described 34.

### In vitro differentiation assays

Before plating onto tissue culture-grade plastic-coated chamber slides (Fisher Scientific, Pittsburgh, PA), APSCs were dissociated by incubation in 0.25% trypsin-EDTA, 1 mg/ml collagenase type 1a (Sigma), 1 mg/ml collagenase IV and 2% hyaluronidase (Sigma) for 5 minutes at 37°C.

Neuroglial differentiation was performed as previously described 35. APSCs were plated in chamber slides in DMEM-F12 (3:1) supplemented with 40 ng/ml bFGF and 10% FBS and were then incubated for 5-7 days. Cells were then cultured for an additional 5-7 days in the same medium without bFGF but with the addition of 10 ng/ml nerve growth factor, 10 ng brain-derived neurotrophic factor (Peprotech) and 10 ng/ml NT3 (Peprotech).

For adipocyte differentiation 36, cells were cultured in medium containing low-glucose DMEM, 1x ITS (Sigma), 1 mg/ml LA-BSA (Sigma), 1 mM hydrocortisone (Sigma), 60 mM indomethacin (Sigma), 0.5 mM isobutylmethylxanthine (Sigma) and 10% horse serum (Invitrogen). To assess adipogenic differentiation, cells were stained for 10 min at room temperature with 0.3% oil red O (Sigma) as an indicator for intracellular lipid accumulation and were counterstained with hematoxylin. For chondrocyte differentiation 36, cells were cultured in medium containing 90% high-glucose DMEM, 10% FBS, 1x ITS, 1 mg/ml LABSA, 50 nM dexamethasone and 60 pM transforming growth factor-β1 (TGF-b1) (R&D Systems). Alcian Blue/Sirius red staining (Sigma) was carried out by applying 0.5% Alcian Blue 8GX for proteoglycan-rich cartilage matrix and 1% Sirius red F3B for collagenous matrix. Osteogenic differentiation was conducted in confluent monolayer cultures of APSCs grown in high-glucose DMEM containing 10% FCS, 100 U/ml penicillin, 100 mg/ml streptomycin, 50 mg/ml L-ascorbic acid 2-phosphate, 10 mM b-glycerophosphate, and 100 nM dexamethasone 37. Osteogenesis was determined using alizarin red S staining (1%) to detect calcium mineralization 37. Endothelial cell differentiation to vascular tubes (in phase image) was accomplished by culturing APSCs in EBM (Cambrex) in 24-well plates precoated with Matrigel (300 AL/well; Becton Dickinson) and vascular endothelial growth factor (VEGF, 10 ng/ml, Sigma) for 2-3 days as described previously 38. For hepatocyte induction, cells were grown on collagen I-coated wells for 14 days in DMEM containing 10% FBS, 10x insulin-transferin-selenium (Gibco), 10 nM dexamethasone (Sigma), 100 ng/ml hepatocyte growth factor (HGF, Peprotech) and 50 ng/ml FGF-4 (Peprotech). Glycogen depositions in APSC-differentiated hepatocytes were visualized with a periodic acid Schiff (PAS) stain kit (ScyTek Laboratories) following the manufacturer's protocol. Briefly, fixed (4% PFA, 10 min) cells were oxidized in periodic acid for 10 min, rinsed in H_2_O and treated with Schiff's reagent for 30 min. Cells were then rinsed in H_2_O and counterstained with hematoxylin. Immunocytochemical analysis was performed using specific antibodies against albumin (Bethyl), α1-antitrypsin (Thermo), α-fetoprotein (Dako) and GATA4 (GeneTex) with differentiated hepatocytes.

### Gene silencing with RNA interference

For shRNA experiments, cells were plated into 10 cm^2^ culture dishes at a density of 5 x 10^4^/cm^2^ and infected with Lenti-FOXC1 shRNA (LV-FOXC1-sh, sc-43766-V, Santa Cruz Biotechnology), Lenti-STI-1 shRNA (LV-STI-1-sh, sc-153893-V, Santa Cruz Biotechnology), Lenti-PrP^C^ shRNA (LV-PrP^C^-sh, sc-36319-V, Santa Cruz Biotechnology) and control shRNA (Santa Cruz Biotechnology) for 48 hours following the manufacturer's instruction. Cell proliferation assays and protein expression of FOXC1, STI-1 and PrP^C^, as determined by western blot, were examined for LV-FOXC1-sh-, LV-STI-1-sh- and LV-PrP^C^-sh-mAPSCs.

### Plasmid constructs and transfection

Cells (3.5 × 10^5^ cells/6-cm well) were grown to 80-90% confluence and then transfected with 4 μg of plasmids of constitutively active FOXC1 (pcDNA4-FOXC1) 39; STI-1 (pB-mSTI1-EGFP) provided by Dr. Blatch^40^ and PrP^C^ (pcDNA3.1-PrP) provided by Dr. Tatzelt 41. Transfection was performed using nucleoporation (Nucleofector, Amaza) according to the manufacturer's protocol. Transfection efficiency was approximately 70% as determined using green fluorescent protein, and maximal levels of protein expression were observed between 24 and 48 hours.

### Quantitative reverse transcription-PCR analysis (qRT-PCR)

Total RNA from APSCs and human neural stem cells (NSCs, H9-derived, Gibco) was purified with TRIzol reagent (Invitrogen) and retrotranscribed to cDNA using a reverse transcriptase Kit (Roche). qRT-PCR reactions were performed as previously described 5 by using the primers shown in Table 1 or Taqman assays (code number: Rn00566603_m1 for Gfap and Rn00565046_m1 for Mtap2, Applied Biosystems). The probe signal was normalized to an internal reference. The relative expression level was calculated using transcript levels of beta actin (Actb) as an endogenous reference. Data analysis was done according to the comparative method following the User Bulletin No. 2 (Applied Biosystems).

### Chromatin immunoprecipitation (ChIP) Assay

To demonstrate the binding of FOXC1 protein to the STI-1 promoter (NCBI Accession number: NC_000011.9), a ChIP assay was performed with a commercial kit (Upstate Biotechnology, USA) using the manufacturer's protocol with minor adjustments. FOXC1-overexpressed mAPSCs were produced by transfecting with pcDNA4-FOXC1 by nucleoporation as described above, and formaldehyde was added directly to the culture medium to a final concentration of 1% followed by incubation for 20 min at 37°C as previously described 42. Nuclear lysates were sonicated (Sonic Dismembrator 60, Fisher Scientific) for five 3-s intervals on ice to shear DNA to 0.800-1.2 kb fragments. Chromatin solutions were precleared with salmon sperm/protein. Precleared supernatants were incubated with anti-FOXC1 (Novus Biologicals) or normal IgG (negative control) and rotated overnight at 41°C. DNA-protein complexes were isolated on salmon sperm DNA linked to protein-A agarose beads and eluted with 1% SDS and 0.1 M NaHCO_3_. Cross-linking was reversed by incubation at 65°C for 5 h. Proteins were removed with proteinase K, and DNA was extracted with phenol/chloroform, re-dissolved and PCR-amplified with STI-1 promoter primers (S1 primers, PCR product 153 bp), sense 5'-GTTTATAGAGGAGCGCCCAA-3' and antisense 5'-GGTCGAGTTCTTCTA GGGGG-3' and negative control primers (S2 primers, PCR product 151 bp), sense 5'-AGCACAGACATTCCCCCCTA-3' and antisense 5'-CCGGAACCCCGTTGAATCGA-3'.

### Generation of promoter constructs and reporter gene assays

A fragment containing the 5'-flanking region (~2000 bp) of the human STI-1 gene promoter (NCBI Accession number: NC_000011.9) was generated from human genomic DNA by PCR. This product was cloned into the BamHI and XbaI sites of the pGL3-basic vector (Promega, USA), which contained one putative FOXC1-responsive element (FRE) (5'-**CAAGCAAATA**-3', -181 to -172), and the generated plasmid was designated pSTI-1-luc1. One additional STI-1 promoter construct (pSTI-1-luc2) using the same downstream primer as for pSTI-1-luc1 did not contain the FRE. In the pSTI-1-mutFRE construct, the putative FRE of pSTI-1-luc1 was replaced from 5'-** CAAGCAAATA**-3' to 5'-**GCGGGTAGGT**-3' using the QuikChange Site-Directed Mutagenesis Kit (Stratagene, USA). All constructs were verified by DNA sequencing. NIH3T3 cells at approximately 90% confluence in 24-well plates were transiently co-transfected with reporter plasmid (0.5 μg) and FOXC1 expression vector (pcDNA4-FOXC1) using jetPEI^TM^ reagent (Polyplus-Transfection, USA) according to the manufacturer's directions. To correct for variable transfection efficiency, cells were cotransfected with the pRL-SV40 vector (0.05 μg) encoding the *Renilla* luciferase gene. Luciferase activity of cell lysates was determined with a multiwell luminescence reader (Molecular Devices, USA) using the Dual-Luciferase Reporter Assay System (Promega, USA).

### Total protein extraction, western blotting and ELISA

mAPSCs were allowed to grow until 80% confluence. Before the addition of stimuli, mAPSCs were washed in phosphate-buffered saline (PBS) and then incubated in the presence or absence of recombinant human STI-1 (Novus Biologicals). Pharmacological inhibitors of PD98059 (p-ERK1/2 inhibitor), SB203580 (p-p38 inhibitor), SP600125 (p-JNK inhibitor), LY294002 (p-Akt inhibitor), and AG490 (p-Jak2 inhibitor) were diluted in dimethylsulfoxide (DMSO); all were purchased from Sigma. All cultures received the same amount of solvent serving as controls. After treatment, cells were rinsed in cold PBS and immediately used for protein extraction.

Western blot analyses of mAPSCs were performed after each treatment as previously described 43. Cells were lysed in a buffer containing 320 mM sucrose, 5 mM HEPES, 1 μg/mL leupeptin, and 1 μg/mL aprotinin. Lysates were centrifuged at 13,000 *g* for 15 min. The resulting pellet was resuspended in sample buffer (62.5 mM Tris-HCl, 10% glycerol, 2% SDS, 0.1% bromophenol blue, and 50 mM DTT) and subjected to SDS-polyacrylamide gel (4-12%) electrophoresis. The gel was then transferred to a Hybond-P nylon membrane. This was followed by incubation with appropriately diluted antibodies to FOXC1 (1:200, Novus Biologicals), STI-1 (1:250, Santa Cruz), PrP^C^ (1:300, Santa Cruz), Proliferative cell nuclear antigen (PCNA, 1:200, Sigma), Bmi-1 (1:300, Chemicon), cdk4 (1:100, Santa Cruz), cyclinD1 (1:250, Chemicon), JunB (1:200, Chemicon), PTEN (1:200, Chemicon), p-ERK1/2, ERK1/2 (1:200-1:300; Cell Signaling), p-JNK, p-p38, JNK and p38, Jak2 (1:200; Santa Cruz), p-Akt and Akt (1:100, Calbiochem), Bcl-2 (1:200; Santa Cruz), Bcl-xL (1:200; Transduction Laboratories), Bax (1:200; Santa Cruz), Bad (1:200; Transduction Laboratories) and β-Actin (1:2000, Santa Cruz). Membrane blocking, primary and secondary antibody incubations, and chemiluminescence reactions were conducted for each antibody individually according to the manufacturer's protocol. The intensity of each band was measured using a Kodak Digital Science 1D Image Analysis System (Eastman Kodak). In addition, the total amount of STI-1 (ELISA kit, USCN) in the medium was measured according to the manufacturer's instructions. Optical density was measured using a spectrophotometer (Molecular Devices), and standard curves were generated with the program SOFTmax (Molecular Devices).

### Knockout mouse lines

A colony of each mouse line was maintained in the animal facility of the China Medical University, Taiwan according to the Institutional Animal Care guidelines. Mice heterozygous for the null mutation *FOXC1^lacZ^*(*FOXC1^+/-^*) were a kind gift from Dr. Kume 17. Timed embryos were obtained by examining for vaginal plugs in mated females. The morning of plug detection was designated E0.5. The homozygous embryos (*FOXC1^-/-^* mice) were genotyped at E12-14.5 according to the procedure established for PCR using primers as described 1, 17, 44, 45. The Ethical Committee for Animal Research at China Medical University has reviewed and approved all animal experiments.

### Immunohistochemical assessment

Embryos were harvested from pregnant dams after chloral hydrate (0.4 g/kg, ip) anesthesia and cervical dislocation. Whole embryonic heads were fixed overnight at 4^o^C in 4% paraformaldehyde (PFA), cryoprotected, and frozen in OCT (Sakura Finetek). Cryosections of 12 μm were cut and stored at -80^o^C until needed. Adult animals were anesthetized with chloral hydrate (0.4 g/kg, ip) and their brains fixed by transcardial perfusion with saline followed by perfusion and immersion with 4% paraformaldehyde. Finally, the brain samples were dehydrated in 30% sucrose. After brains were frozen on dry ice, a series of adjacent 6-μm-thick sections were cut in the coronal plane with a cryostat, stained with H&E and observed by light microscopy (Nikon, E600). Blue color fluorescence (from bisbenzimide) of brain sections was detected directly by fluorescence microscopy (Carl Zeiss, Axiovert 200M, Germany).

### Laser-scanning confocal microscopy for immunofluorescence co-localization analysis

To demonstrate the differentiation potential of transplanted cells, the expression of cell type-specific markers in bisbenzimide-labeled APSCs was identified by immunofluorescence colocalization analysis of each brain section. Cell type-specific markers such as glial fibrillary acidic protein (GFAP), von Willebrand factor (vWF), microtubule-associated protein 2 (MAP-2) and βIII-tubulin (Tuj-1) were stained to determine if they colocalized with bizbenzimide in the same cell. Additionally, each coronal section was subsequently immunoassayed with primary antibodies: glial fibrillary acidic protein (GFAP for astrocytes, 1:400, Sigma), microtubule-associated protein 2 (MAP-2 for neuronal dendrites, 1:200; BM), βIII-tubulin (Tuj-1, 1:200, Chemicon), Von-Willebrand factor (vWF for endothelial cells, 1:20, Santa Cruz Biotechnology), FOXC1 (1:200, Novus Biologicals), STI-1 (1:250, Santa Cruz Biotechnology), PrP^C^ (1:300, Santa Cruz Biotechnology), nestin (1:300, Millipore), NGFRp75 (1:100, Millipore), GFAP (1:400, Sigma Aldrich), activated caspase-3 (1:200, Chemicon) and Ki67 (1:300, Chemicon) conjugated with FITC or Cy-3 (1:500, Jackson ImmunoResearch PA). The tissue sections were analyzed in 3D images with a Carl Zeiss LSM510 laser-scanning confocal microscope. The total number of cells co-stained with bisbenzimide and cell type-specific markers was measured as previously described 19.

### Brain explant

On day E13.5, the pregnant mothers were injected with BrdU (50 mg/kg, Sigma) and the fetal brains were collected 1 hour later. The brain explants, with intact meninges, were embedded in low-melt agarose (3% SeaPlaque, Cambrex) and cut into 300-μm coronal sections with a vibratome (Leica). For the brain explant co-culturing experiments, DMEM/F12 media containing 10% fetal bovine serum, 2 mM glutamine, 100 mM dextrose, 100 μM penicillin/streptomycin was removed 2 days prior to the explant experiment and replaced with 1.5 ml of Neurobasal (NB) medium (Invitrogen) containing B27 supplement with or without recombinant human STI-1 (100 ng/mL, Novus Biologicals). Thus, the media was conditioned for 48 hours prior to the explant experiment. Filter inserts (0.40 μm, Millipore) in 60-mm plates (Costar) were placed in the wells containing the mAPSCs/conditioned media (mAPSCs-CM), and NL (control) or *FOXC1^-/-^* brain explants were transferred into the inserts and cultured for 36 hours post BrdU injection. For treatment with STI-1, a stock solution of 1 mg/mL STI-1 was diluted to 100 ng/mL in NB medium. Following culturing, all explants were fixed for 30 min in 4% paraformaldehyde and cryosectioned in 10-μm increments. Sections were immunolabeled for BrdU and Ki-67 and analyzed for cell cycle exit as described above.

### Isolation of mouse neural stem cells (mNSCs) and neurospheres

The mouse brains of various stages were prepared from *FOXC1^-/-^, FOXC1*^+/-^ and normal littermate (NL) mice. Embryonic subventricular zone (SVZ) cells (E14.5) were aseptically isolated and dissociated as previously described 46, 47. Then, mNSCs forming neurospheres were obtained in DMEM/F12 (Gibco) supplemented with 1% B27 (Invitrogen), 2 mM L-glutamine (PAN), and 100 U/ml penicillin / 0.1 mg/L streptomycin (Gibco). For maintenance and expansion of the cultures, the DMEM/F12 was further supplemented with 1% N2 supplement, 1% B27 (Invitrogen), 2 μg/mL heparin (Sigma), 20 ng/mL FGF-2 (R&D Systems) and 20 ng/mL EGF (R&D Systems). Cultures were maintained at 37°C in a humidified incubator with 5% CO_2_. Neurosphere cultures from passage number 4 to 6 were used in this study. For the neurosphere co-culturing experiments, DMEM/F12 media containing 1% B27 (Invitrogen), 2 μg/mL heparin (Sigma), 20 ng/mL FGF-2 (R&D Systems) and 20 ng/mL EGF (R&D Systems) was removed and replaced with 1.5 ml of Neurobasal (NB) medium (Invitrogen) containing B27. Filter inserts (0.40 μm, Millipore) in 60-mm plates (Costar) were placed in the wells containing the mAPSCs/conditioned media (mAPSCs-CM), and NL (control) or *FOXC1^-/-^* NPCs were transferred into the inserts and cultured for 36 hours post BrdU injection. For treatment with STI-1, a stock solution of 1 mg/mL STI-1 was diluted to 100 ng/mL in NB medium. Following culturing, all explants were fixed for 30 min in 4% paraformaldehyde and cryosectioned in 10-μm increments. Sections were immunolabeled for BrdU and Ki-67 and analyzed for cell cycle exit as described above.

### Preparation of lentiviral constructs of STI-1

The lentiviral constructs were generated by co-transfection of human kidney-derived 293T cells with three plasmids using the calcium phosphate method as previously described 48. In brief, in this transducing vector, the lentiviral vector pLKO AS2 (National RNAi Core Facility, Taiwan) served as the backbone to generate a lentiviral vector used to direct the expression of STI-1-EGFP cloned from the above plasmid of pB-mSTI1-EGFP (LV-STI-1) and GFP (LV-GFP). The third plasmid provides the envelope protein from the vesicular stomatitis virus glycoprotein to enhance viral stability and broaden the range of host cell types. The virus was harvested by collecting the cell culture medium after 48 h. After filtering the collected medium through 0.45-mm filters, the virus was concentrated by spinning at 4000 g for 15 min followed by a second spin (1000 g, 2 min at room temperature). The concentrated virus was stored at -80^o^C.The titer of lentiviral vectors was determined by dilution. The lentiviral titers were determined by infection of 293T cells that were seeded in six-well plates at 1 × 10^5^ cells per well on the day before infection with serial dilution of the concentrated viral stock. After overnight incubation, the culture medium was changed, and the cells were incubated for two more days. GFP fluorescent cells were identified by fluorescence microscopy or by a fluorescence-activated cell sorter. Titers ranged from 10^8^ to 10^9^ infectious units (IU)/mL.

### In Vitro primary cortical culture (PCC) preparation

Primary cortical cultures (PCCs) were prepared from the cerebral cortex of gestation day 17 embryos from C57BL/6 mice as described previously (Wetzel M, Cell Death Differ 2008;15:143-151). PCCs were maintained under serum-free conditions in neurobasal medium (Invitrogen) supplemented with B-27 (2%; Invitrogen), glutamine (0.5 mM; Sigma Aldrich), glutamate (25 mM; Sigma Aldrich), penicillin (100 U/ml) and streptomycin (100 mg/ml; Invitrogen). At 4 days in vitro, half of the medium was removed and replaced with fresh medium without glutamate as indicated by the manufacturer. The cultures were maintained in a humidified incubator at 37°C with 5% CO2. At 7 days in vitro, PCCs were used for experimentation.

### Co-cultures of APSCs with PCCs and oxygen-glucose deprivation (OGD)

For direct co-cultures, APSCs (5 × 10^4^) were added to PCCs at a density of 100,000 cells in each well of a 6-well tissue culture plate. Co-cultures were maintained in neurobasal medium with B27. The medium was changed once each week. For oxygen-glucose deprivation (OGD) treatment, the co-cultured cells were incubated with glucose-free Earle's balanced salt solution, placed in a hypoxic chamber (Bug Box, Ruskinn Technology, UK) for 4 hours and continuously flushed with 95% N_2_ and 5% CO_2_ at 37°C to maintain a gas phase PO_2_ of < 1 mm Hg (OM-14 oxygen monitor; SensorMedics). Control cells were incubated in glucose-free Earle's balanced salt solution in a normoxic incubator for the same period. OGD was terminated by switching to normal culture conditions.

### Assessment of neurite regeneration in vivo and in vitro

Brain tissue samples and PCCs were immunostained to measure neurite outgrowth as described earlier 49. Briefly, brain tissue samples and PCCs were fixed and immunostained with specific antibody against βIII-tubulin (Tuj-1, 1:400; Chemicon). For quantification, neurons with processes greater than twice the cell body diameter were counted as neurite-bearing cells. The length of the longest neurite in each neuron was measured in digitized images and quantified using image analysis software (SigmaScan).

### Animal brain ischemia/reperfusion model

Adult male Sprague-Dawley (SD) rats (weight 250-300 g) were used in this study. Animals were subjected to three-vessel ligation. All surgical procedures were performed by sterile/aseptic techniques in accordance with our institutional guidelines. The rats were anesthetized with chloral hydrate (0.4 g/kg, ip). Ligation of the right middle cerebral artery (MCA) and bilateral common carotids (CCAs) was performed by methods described previously with slight modifications 50. The bilateral CCAs were clamped with non-traumatic arterial clips. Using a surgical microscope, a 2 × 2 mm craniotomy was drilled where the zygoma fuses to the squamosal bone. The right MCA was ligated with 10-0 nylon suture. Cortical blood flow was measured continuously with a laser Doppler flowmeter (PF-5010, Periflux system, Perimed AB, Stockholm, Sweden) in anesthetized animals. A burr hole (1 mm diameter) was made in the right frontoparietal region to allow placement of photodetectors. A probe (0.45 mm in diameter) was stereotaxically placed in the cortex (1.3 mm posterior, 2.8 mm lateral to the bregma, and 1.0 mm below the dura). After 90 minutes of ischemia, the suture on the MCA and arterial clips on CCAs were removed to allow reperfusion. Core body temperature was monitored with a thermistor probe and maintained at 37°C with a heating pad during anesthesia. After recovery from anesthesia, body temperature was maintained at 37°C with a heat lamp 43.

### Stereotaxic transplantation of APSCs

Prior to transplantation, the APSCs and human neural stem cells (NSCs, Gibco) were labeled using 1 μg/mL bisbenzimide (Hoechst 33342; Sigma) for 1 hour at 37°C as previously described 43. Labeled cells were then collected and washed in PBS three times. APSCs were counted using a cytometer to ensure an adequate cell number for transplantation. One week after brain ischemia, adult male Sprague-Dawley rats (weight > 300 g) were anesthetized with chloral hydrate (0.4 g/kg, ip) and injected stereotaxically with approximately 1 × 10^6^ cells in a 3-5 μL PBS suspension through a 26-gauge Hamilton syringe into 3 cortical areas adjacent to the right MCA, 3.0 to 5.0 mm below the dura. The control animals were administered PBS only. The approximate coordinates for these sites were 1.0 to 2.0 mm anterior to the bregma and 3.5 to 4.0 mm lateral to the midline, 0.5 to 1.5 mm posterior to the bregma and 4.0 to 4.5 mm lateral to the midline, and 3.0 to 4.0 mm posterior to the bregma and 4.5 to 5.0 mm lateral to the midline. The needle was retained in place for 5 minutes after each injection and a piece of bone wax was applied to the skull defects to prevent leakage of the injected solution. For blocking experiments, stereotaxic injection of LV-STI-1-sh- or LV-LV-PrP^C^-sh-treated APSCs was performed with the stroke rats.

### Neurological behavioral assessment

Behavioral assessments were performed 5 days before cerebral ischemia and 1, 7, 14 and 28 days after cell transplantation. The tests measured body asymmetry and locomotor activity. The baseline test scores were recorded in order to normalize those taken after cerebral ischemia. The elevated body swing test was used to assess body asymmetry after MCA ligation and evaluated quantitatively as previously described 19. Initially, animals were examined for lateral movement with their bodies suspended by their tails 10 cm above the cage floor. The frequency of initial head swing contra-lateral to the ischemic side was counted in twenty continuous tests and was normalized to the baseline score. For locomotor activity measurements, rats were subjected to VersaMax Animal Activity Monitoring (Accuscan Instruments, Inc., Columbus, OH) for approximately 2 hours for behavioral recording 19. This instrument contained 16 horizontal and 8 vertical infrared sensors. The vertical sensors were positioned 10 cm from the floor of the chamber. Motor activity was determined as the number of beams broken by a rat's movement in the chamber. Three parameters of vertical activity during 2 hours were calculated, including (i) vertical activity, (ii) vertical time, and (iii) number of vertical movements. Furthermore, grip strength was analyzed using the Grip Strength Meter (TSE-Systems, Germany) as previously described with modifications 19. In brief, the grip strength ratio of each forelimb was measured separately and was calculated as the ratio of the mean strength of 20 pulls of the side contralateral to the ischemia relative to that of the ipsilateral side. In addition, the ratio of grip strength post-treatment and pre-treatment was also calculated, and the changes are shown as a percentage of the pre-treatment value.

### [^18^F]fluoro-2-deoxyglucose positron emission tomography (FDG-PET) examination

To assess the metabolic activity and synaptic density of brain tissue, experimental rats were examined using microPET scanning of [^18^F]fluoro-2-deoxyglucose (FDG) to measure relative metabolic activity following the protocol previously described 51. In brief, ^18^F was produced by the ^18^O(p, n)^18^F nuclear reaction in a cyclotron at China Medical University and Hospital, Taichung, Taiwan, and ^18^F-FDG was synthesized as previously described 52 with an automated ^18^F-FDG synthesis system (Nihon Kokan, Tokyo, Japan). Data were collected with high-resolution small-animal PET (microPET, Rodent R4, Concorde Microsystems Inc., Knoxville, TN). The system parameters have been described previously by Carmichael et al. 53. After one week of each treatment, animals were anesthetized with chloral hydrate (0.4 g/kg, ip), fixed in a customized stereotactic head holder and positioned in the microPET scanner. Then, the animals were given an intravenous bolus injection of ^18^F-FDG (200-250 μCi/rat) dissolved in 0.5 ml of saline. Data acquisition began at the same time and continued for 60 min using a 3D acquisition protocol. The image data acquired from microPET were displayed and analyzed by IDL ver. 5.5 (Research Systems, Colorado, USA) and ASIPro ver. 3.2 (Concorde Microsystems Inc., Knoxville) software. FDG-PET images were reconstructed using a posterior-based 3D iterative algorithm 54 and overlaid on MR templates to confirm anatomical location 55. Coronal sections for striatal and cortical measurements represented brain areas between 0 and +1 mm from bregma and for thalamic measurements between -2 and -3 mm from bregma as estimated by visual inspection of the unlesioned side. The relative metabolic activity in regions of interest (ROIs) of the striatum was expressed as a percentage deficit as previously described with modifications 53.

### Statistical analysis

All measurements in this study were performed blindly. The results are expressed as the mean ± SEM. The behavioral scores were evaluated for normality. We used two-way ANOVA with appropriate post hoc Newman-Keuls testing to evaluate the mean differences between different groups. A value of *P* < 0.05 was taken as significant.

## Results

### Isolation and expansion of arachnoid-pia-derived spheres from adult human and mouse meninges

**Figure 1:**
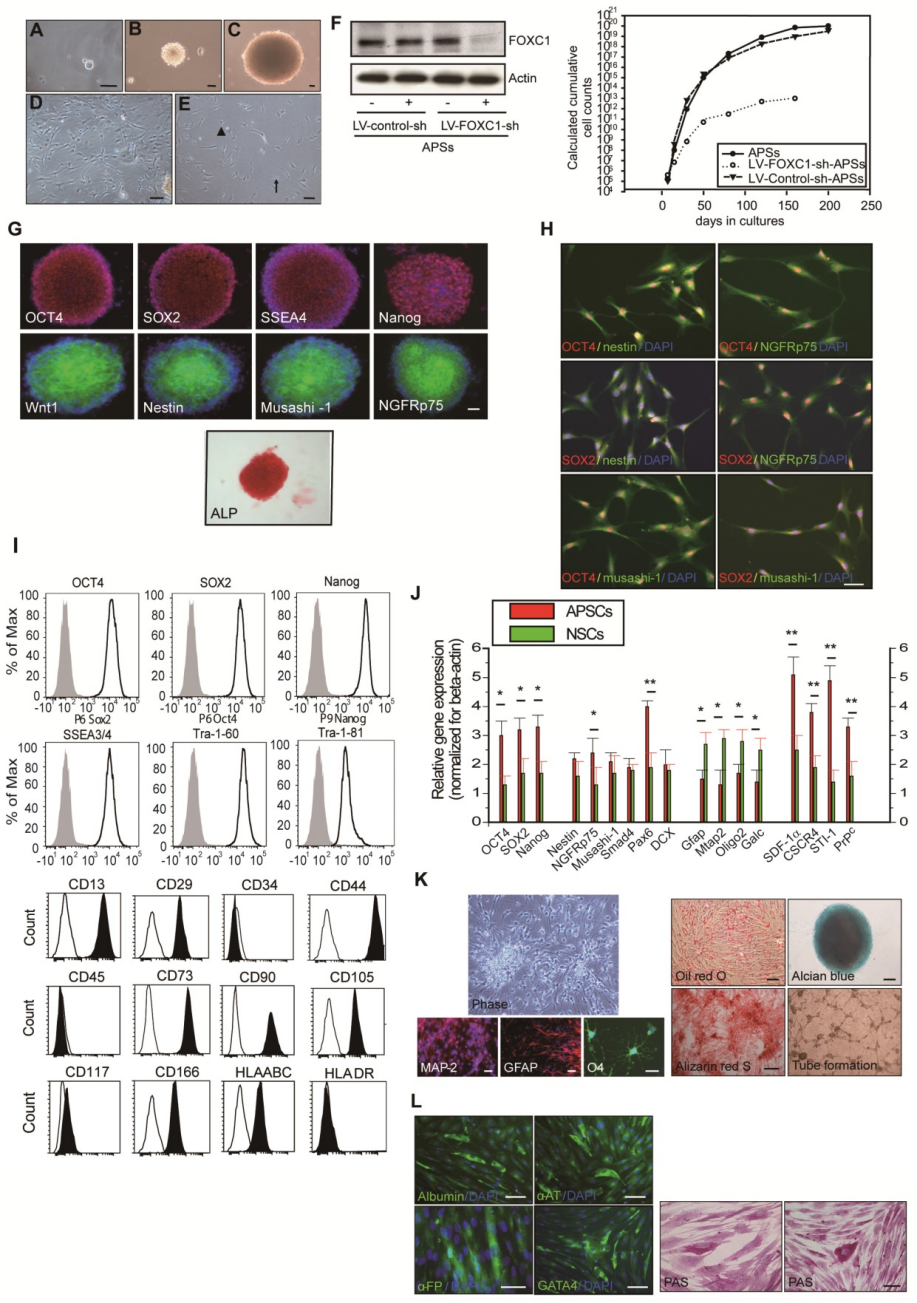
** Isolation and characterization of arachnoid-pia stem cells (APSCs) from adult human meninges.** (**A**) Representative images of APSCs at 5-7 days; some cell clusters started to form in the primary culture of arachnoid-pia tissues from adult human meninges. (**B-C**) After 10-14 days, 3-dimensional (3D) arachnoid-pia spheres (APSs) adhered to plastic wells, and only a small number of spheres floated. (**D-E**) Many neuroglia-like cells migrated from the spheres and show a unique morphology (phase contrast). (**F**) In western blotting, FOXC1 expression was decreased by lenti-FOXC1-sh transduction in APSs. The growth kinetic analysis data showed a reduction of proliferative cell numbers in the LV-FOXC1-sh-APSs. (**G**) In immunocytochemistry (IHC), a neural crest cell marker (Wnt1), four ES cell markers (OCT4, SOX2, SSEA4 and Nanog) and positive ALP staining were consistently expressed in most of the APSs. Representative NSC markers of nestin, musashi-1 and NGFRp75 were also strongly immunoreactive. (**H**) Using a double immunofluorescence study, co-staining of nestin^+^, musashi-1^+^ or NGFRp75^+^ cells was observed with markers of OCT4 or SOX2 in the adhesive neuroglia-like cells. (**I**) Flow cytometric analysis in isolated cells from APSCs was positive for ES cell markers and mesenchymal stem cell origin but negative for hematopoietic stem cell origin. (**J**) Relative gene expression analysis of APSCs and human NSCs (n=3 independent experiments). For each sample, expression levels of different genes were normalized to the levels of b-actin mRNA. (**K**) In *in vitro* differentiation assays, APSCs differentiated into neuroglial cell morphology (phase for neural phenotype and IHC for MAP-2, GFAP and O4 in the upper panel), mesenchymal phenotypes (adipocyte: oil-red-O staining; chondrocyte: alcian blue staining; and osteocyte: alizarin red staining in the lower panel) and endothelial cells (tube formation-phase contrast). (**L**) In endodermal differentiation, immunocytochemical analysis of differentiated hepatocytes was positive for human albumin, α-feto-protein (α-FP), α1-antitrypsin (α1-AT) and GATA4. Glycogen storage in the hepatocyte was examined by PAS staining. Data are expressed as the mean ± SEM. * *P* < 0.05 and * *P* < 0.01 vs. control, Bar = 40 μm

In preparing the primary culture of arachnoid-pia tissues from the adult human meninges, although most of the single cells gradually died, some cells started forming clusters within 5-7 days of culture (Figure 1A). After 10-14 days, characteristic 3-dimensional (3D) spheres were observed (Figure 1B-C), which were termed “arachnoid-pia spheres” (APSs). The majority of APSs adhered to plastic wells (Figure 1D-E), and only a small number of spheres floated. The APSs were easily detached by tapping the flasks as they separated from the monolayer cells, which were strongly attached to the culture flasks. The subcultured spheres showed the same morphology as those in the primary culture (Figure 1B). Twenty-seven of 30 (90%) arachnoid-pia membrane samples formed spheres (~0.1 mm in diameter), and the sphere-forming efficiency of single cells was approximately 0.088% (4400 spheres from 5 × 10^6^ single cells). In addition, some isolated cells that migrated from the spheres exhibited a unique neuroglia-like morphology (Figure 1 D-E). To examine the proliferation rate of APSs regulated by the expression of FOXC1, we first performed protein knockdown by LV-FOXC1-sh transduction in APSs, which revealed significant downregulation of FOXC1 by western blotting (Figure 1F). Significant reduction of proliferative cell numbers in the LV-FOXC1-sh-APSs compared to that of LV-Control-sh-APSs and APSs was demonstrated by the growth kinetic assay (Figure 1F).

To characterize the expression of stem cell markers in APSs, the spheres were examined by immunocytochemistry. In addition to the neural crest cell lineage marker Wnt1, four embryonic stem (ES) cell markers (OCT4, SOX2, SSEA4, and Nanog) and positive alkaline phosphatase (ALP^+^) staining were found to be consistently expressed in most of the APSs (Figure 1G-H). The majority of cells within the APSs were strongly immunoreactive for the neural stem cell markers nestin, musashi-1 and NGFRp75 (Figure 1G). Some of the nestin^+^, musashi-1^+^ or NGFRp75^+^ cells co-expressed with markers of OCT4 or SOX2 (Figure 1H). In flow cytometric analysis, the APSs strongly express ES cell markers (OCT4, SOX2, Nanog, SSEA3/4, Tra-1-60, Tra-1-81) (Figure 1I). Moreover, these cells were negative for CD34, CD45, CD117 and HLA-DA but positive for CD13, CD29, CD44, CD73, CD90, CD105, CD166 and HLA-ABC (Figure 1I). These observations indicated that cells isolated from APSs have the same surface markers as those of mesenchymal stem cell (MSCs) (Figure 1I). In the meantime, adult mouse meninge-derived APSs (AmAPSs) and APSCs (AmAPSCs) was also isolated and characterized to show the similar cellular and biological property as that from adult human meninges (Supplemental Figure 1A-L).

Gene expression analysis was performed to compare the stemness molecular signature and neural differentiation pattern of APSs with that of neurospheres from human NSCs (Figure 1J). APSs showed higher pluripotent gene expression, such as OCT4, SOX2 and Nanog compared with NSCs. A significant difference between NSC and APS cultures occurs for the expression levels of SDF-1α, CXCR4, PrP^C^ and STI-1. Statistically relevant differences were also observed for neural progenitor markers of Nestin, NGFRp75, Musashi-1, Smad4, Pax6, and Dcx. In contrast, APSs expressed lower neural differentiation genes, such as Mtap2, Gfap, oligo2 and Galc, compared with NSCs (Figure 1J). These results indicate that spheres obtained from meninges and from parenchyma are different.

### APSCs possess multipotent differentiation potential

The multipotency of APSCs was characterized by the induction of differentiation into several cell lineages *in vitro*. Re-formed APSs were enzymatically dissociated into single cells and were plated onto coated tissue-culture-grade plastic in differentiation medium. After 2-3 weeks under appropriate differentiation culture conditions, cells were characterized by immunostaining. When cultured under the same conditions that allowed the differentiation of APSCs into neuroglial cells 35, cells acquired a MAP-2^+^ neuronal morphology and expressed GFAP and O4 (Figure 1K).

APSCs were also able to differentiate into chondrocytes as shown by alcian blue staining (Figure 1K) and osteocytes as shown by alizarin red staining (Figure 1K). Adipogenesis was seen when APSCs were cultured in adipocyte differentiation medium. After 2 weeks of culture, lipid droplets were present in some cells, which could be detected by Oil-red-O staining (Figure 1K). In endothelial cell differentiation, APSCs were successfully induced into endothelial cells that exhibited vascular tube formation (Figure 1K). Finally, in hepatocyte induction medium, differentiated cells were immunoreactive for human albumin, α-feto-protein, α1-antitrypsin and GATA4 (Figure 1L). To test for hepatocellular metabolic functions, polyglycans were shown by the PAS staining method, indicating glycogen storage within the cytoplasm of differentiated cells (Figure 1L).

### Maintenance of self-renewal and proliferation in mAPSCs by activating FOXC1

Both FOXC1 and FOXC2 do play early roles in mesoderm development, this may be a better connection for FOXC transcription factors to stem cell function? Since *FOXC1^-/-^* mice show progressive prenatal cerebral abnormalities 17, raising the possibility that FOXC1 regulates stem cell function.

**Figure 2:**
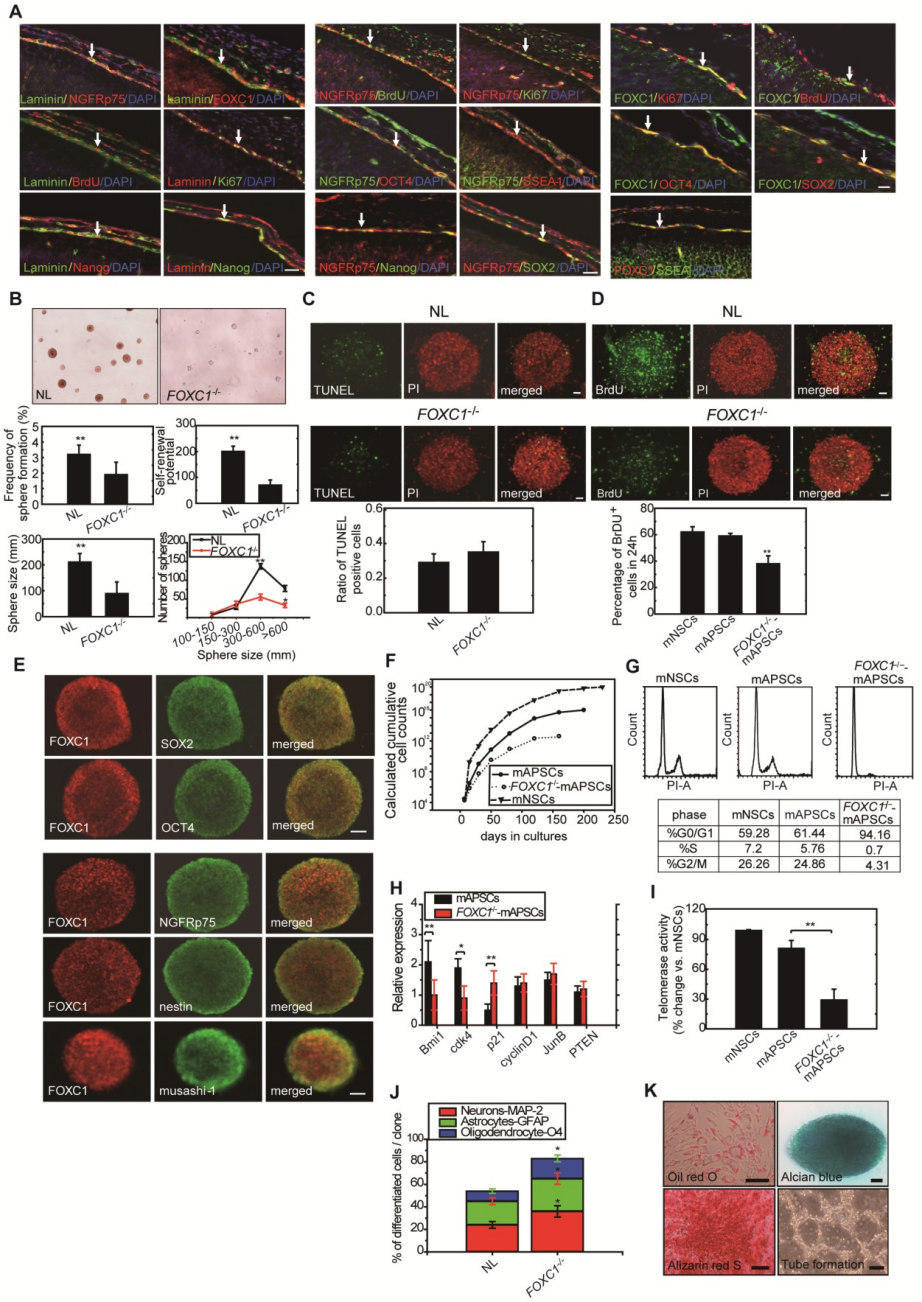
** Proliferation and self-renewal of mAPSCs were modulated by FOXC1 activation.** (**A**) Using immunohistochemistry, laminin^+^NGFRp75^+^ and laminin^+^FOXC1^+^ cells were found in the leptomeningeal layer that covers the brain parenchyma. Both laminin and NGFRp75 immunopositive cells were co-expressed with proliferative markers (BrdU and Ki67) and ES cell markers (OCT4, SOX2, SSEA-1 and Nanog). (**B**) In a representative image of sphere formation, non-adherent cultures of mAPSCs form spheres from *FOXC1^-/-^*mice and their NLs. A lower rate of sphere formation was found in *FOXC1^-/-^*compared to NL cells. Smaller and fewer mAPSs with secondary subcloning were also observed in *FOXC1^-/-^*mice compared to their NLs. In a sphere size analysis, a significant decrease in the number of *FOXC1^-/-^-*mAPSs was found within the 300-600-μm range and >600-μm compared with that in NLs. (**C**) In TUNEL staining, there was no significant difference in cell death between mAPSs from *FOXC1^-/-^* and NL mice. (**D**) In BrdU immunostaining, a significant decrease of proliferation by BrdU incorporation was observed in mAPSs of *FOXC1^-/-^* mice. (**E**) In a double immunofluorescence study of mAPSs, co-expression of FOXC1 was found with the stem cell markers OCT4, SOX2, nestin, NGFRp75 and musashi-1. (**F**) In growth kinetic analyses, an exponentially increasing pattern in mAPSCs was comparable to that in mNSCs. In contrast, *FOXC1^-/-^-*mAPSCs show a reduction of the self-renewal potential. (**G**) In an examination of the cell-cycle profile by flow-cytometry, a higher percentage of mAPSCs in the S/G2-M phases of the cell cycle was found compared to that of *FOXC1^-/-^*-mAPSCs (mNSCs as a positive control). (**H**) Western blots exhibited significant upregulation of Bmi1 and cdk4 as well as downregulation of p21 in mAPSCs compared to that of *FOXC1^-/-^*-mAPSCs, but no difference in cyclinD1, JunB and PTEN. (**I**) Higher telomerase activity was found in mAPSCs than in *FOXC1^-/-^*-mAPSCs (mNSCs as a positive control). (**J**) The bar chart indicates that enhancement of mAPSC differentiation to neuroglial cells was observed in *FOXC1^-/-^*mice compared to their NLs. (**K**) In *in vitro* differentiation assays, mAPSCs differentiated to mesenchymal phenotypes (adipocyte: oil-red-O staining; chondrocyte: alcian blue staining; and osteocyte: alizarin red staining) and endothelial cells (tube formation-phase contrast). Data are expressed as the mean ± SEM. * *P* < 0.05 and ** *P* < 0.01 vs. control, Bar = 40 μm

To determine whether FOXC1 regulates self-renewal of mAPSCs, we examined the effect of FOXC1 deficiency on the self-renewal of mAPSCs at embryonic day 14.5 (E14.5). First, using immunohistochemistry, we identified the flattened cells adherent to a continuous laminin^+^ leptomeningeal layer that covers the brain parenchyma. The laminin^+^ arachnoid-pia cells co-expressed progenitor markers of NGFRp75 and the transcriptional factor of FOXC1 (Figure 2A and Supplemental Figure 2). We demonstrated that both laminin and NGFRp75 expression co-localized with proliferative markers (BrdU and Ki67) and ES cell markers (OCT4, SOX2, SSEA-1 and Nanog) in the arachnoid-pia membrane of NLs at E14.5 (Figure 2A and Supplemental Figure 2). mAPSCs can form spheres in non-adherent cultures (Figure 2B), and *FOXC1^-/-^* mice forebrain meningeal cells formed multipotent neurospheres (mAPSs) but at a significantly lower frequency (P < 0.05) than did normal littermate (NL) cells (Figure 2B). This suggests that there are significantly fewer stem cells in the embryonic *FOXC1^-/-^* mouse brain. The *FOXC1^-/-^* mAPSs were also approximately 8-fold smaller in sphere numbers (*P* < 0.01) and gave rise to 25-fold fewer (*P* < 0.01) secondary spheres on subcloning, indicating a defect in self-renewal (Figure 2B). Thus, loss of FOXC1 reduced cell proliferation and self-renewal of mAPSCs.

In a study of sphere size, mAPSs from *FOXC1^-/-^* mice (E 14.5) were found to be smaller than those of NLs (Figure 2B). Size analysis of *FOXC1^-/-^-*mAPSs showed a significant decrease in the number of spheres within the 300-600-μm range and >600-μm compared with that in NLs (Figure 2B). In TUNEL assay, there was no increase in cell death in mAPSs from *FOXC1^-/-^* or NL mice (Figure 2C). In contrast, the proliferation of mAPSs of *FOXC1^-/-^* mice assessed by BrdU incorporation was significantly reduced (Figure 2D). Thus, the reduced self-renewal of *FOXC1^-/-^*-mAPSs in the embryonic stage was caused at least partly by reduced proliferation.

To investigate FOXC1 regulation of mAPSC self-renewal, a double immunofluorescence study was carried out. In this study, FOXC1 was co-expressed with specific markers of OCT4, SOX2, nestin, NGFRp75 and musashi-1 (Figure 2E). mAPSCs appeared to rapidly divide and extensively expand in an exponentially increasing pattern, which was comparable to the pattern seen in mouse neural stem cells (mNSCs as a positive control) (Figure 2F). Moreover, growth kinetic analyses in mAPSCs revealed that these cells doubled every 39 hours and could be maintained in culture for more than 200 days without signs of senescence and spontaneous differentiation (data not shown). The self-renewal potential as seen in the growth kinetic analysis was reduced in *FOXC1^-/-^*-mAPSCs (Figure 2F).

We next determined the impact of FOXC1 deficiency on the rate of mAPSC proliferation by analyzing the cell-cycle profile of mAPSCs. Loss of FOXC1 disrupts the cell cycle progression, causing an increase in G0/G1 cells and a concomitant decrease in S and G2/M cells as indicated by the DNA content of the cells. Propidium iodine (PI) staining for DNA content of mAPSCs revealed a 2-fold increase in the percentage of mAPSCs in the S/G2-M phases of the cell cycle when compared with *FOXC1^-/-^-*mAPSCs (mNSCs as a positive control) (Figure 2G).

To investigate the molecular mechanisms underlying the enhanced proliferation of mAPSCs compared to *FOXC1^-/-^-*mAPSCs, we next examined the expression of several genes previously implicated in the self-renewal function. In western blot analysis, *FOXC1^-/-^-*mAPCS had significantly reduced expression of Bmi1 and Cdk4 and increased expression of cell cycle inhibitor protein p21 (Figure 2H). These data demonstrated that FOXC1 expression in mAPSCs is controlled in a cell-cycle-dependent manner and suggested derepression of Bmi1 and cdk4 and reduced expression of p21 as likely molecular mechanisms underlying the enhanced proliferative activity of mAPSCs.

To examine whether the telomerase activity is correlated to the stemness of mAPSCs, we analyzed and showed that telomerase activity in mAPSCs was similar to mNSCs; however, mAPSCs showed higher telomerase activity than that of *FOXC1^-/-^*-mAPSCs (Figure 2I).

Interestingly, the capacity for multilineage differentiation such as MAP-2^+^, GFAP^+^ astrocytes and O4^+^ oligodendrocytes was significantly enhanced in the *FOXC1^-/-^*-mAPSs compared to that of NLs (Figure 2J). mAPSCs were also able to differentiate into chondrocytes as shown by alcian blue staining (Figure 2K) and osteocytes as shown by alizarin red staining (Figure 2K). Adipogenesis was seen when mAPSCs were cultured in adipocyte differentiation medium. After 2 weeks of culture, lipid droplets were present in some cells, which could be detected by Oil-red-O staining (Figure 2K). In endothelial cell differentiation, mAPSCs were successfully induced into endothelial cells that exhibited vascular tube formation (Figure 2K). Taken together, these data indicate that FOXC1 plays a critical role not only in mAPSC proliferation but also in self-renewal potential and differentiation.

### FOXC1 activates the STI-1 promoter in mAPSCs

**Figure 3:**
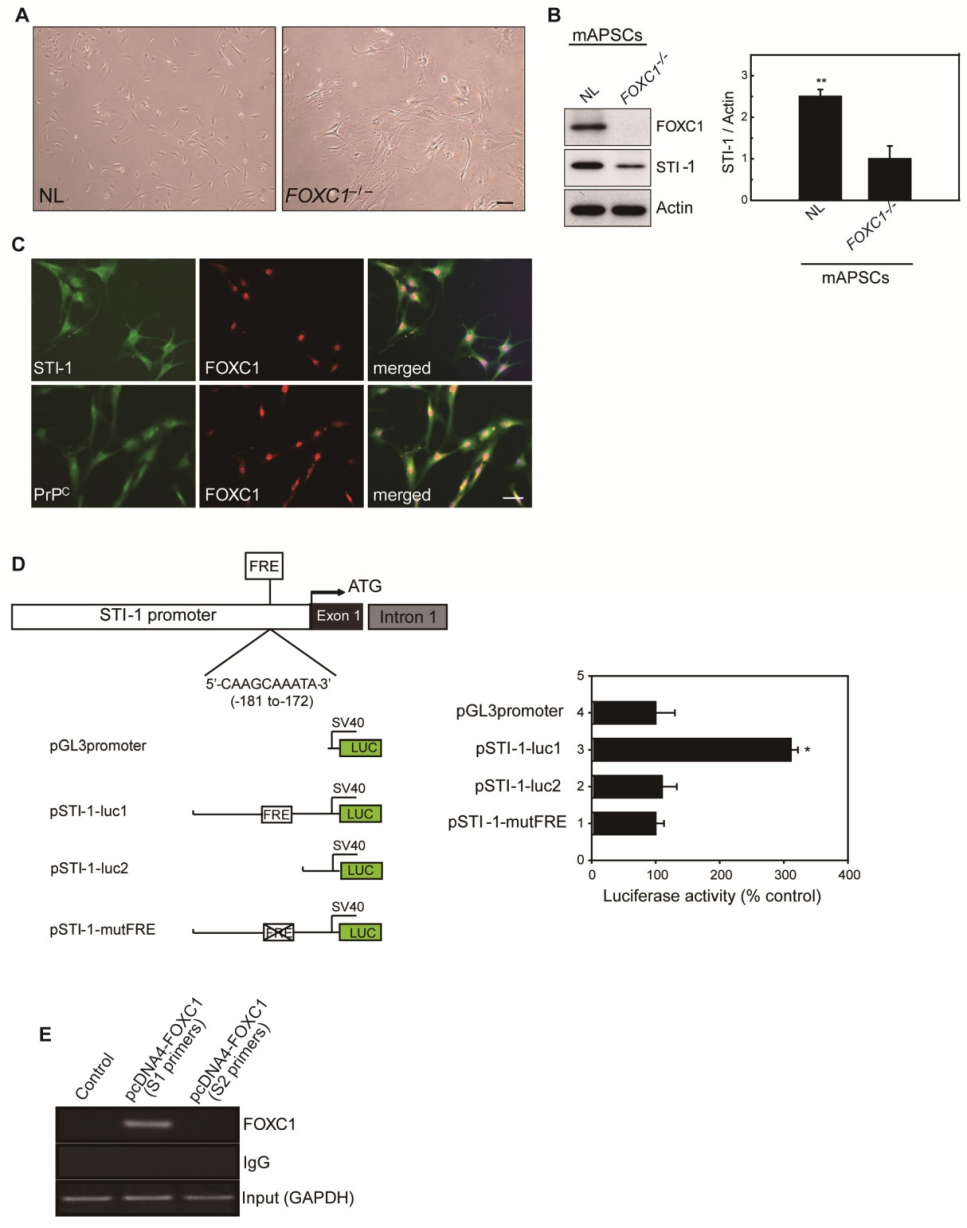
** FOXC1 regulated STI-1 expression in mAPSCs.** (**A**) Representative images of cell morphology in NL- and *FOXC1^-/-^*-mAPSCs. (**B**) Focusing on expression of STI-1 in APSs shown in Figure 1I, western blotting further confirms increased expression of FOXC1-targeted genes of STI-1 in mAPSCs compared to *FOXC1^-/-^*-mAPSCs. (**C**) In a double immunofluorescence study, mAPSCs co-expressed FOXC1 with STI-1 and PrP^C^. (**D**) Schematic representation of FRE (5'-**AAGCAAATA**-3') in the STI-1 promoter (-180 to -172). In a luciferase reporter assay, overexpression of FOXC1 significantly activated luciferase expression with the reporter pSTI-1-luc1 containing the FRE, but not with the reporter without FRE (pSTI-1-luc2) and with a mutation of the putative FOXC1-binding site (pSTI-1-mutFRE). (**E**) In ChIP assays, overexpressed FOXC1 protein from pcDNA4-FOXC1-mAPSCs bound to the endogenous promoter region of STI-1 containing the FRE (using specific primers of S1, but not control primers of S2). Data are expressed as the mean ± SEM. * *P* < 0.05 and ** *P* < 0.01 vs. control, Bar = 40 μm

Because of the close interaction between CXCR4 and PrP^C^ [19]and relevant gene expression analysis results in Figure 1L, we were particularly interested in further characterizing stress-inducible protein 1 (STI-1) because of the critical involvement of the STI-1/PrP^C^ signaling complex in the migration and proliferation of stem cells 20. To demonstrate the difference between NL-mAPSCs and *FOXC1^-/-^*-mAPSCs, cellular phenotype and protein expression pattern were analyzed. More senescent and enlarged cell morphology in *FOXC1^-/-^*-mAPSCs were observed than that in NL-mAPSCs (Figure 3A). Importantly, an increased expression of STI-1 was found in NL-mAPSCs compared to *FOXC1^-/-^*-mAPSCs by western blotting (Figure 3B). Double immunostaining confirmed the co-expression of FOXC1 with STI-1 and PrP^C^ in mAPSCs (Figure 3C).

To evaluate the direct involvement of FOXC1 in the induction of STI-1 expression at the transcriptional level, we first generated a luciferase reporter containing a 2-kb region upstream of STI-1 (pSTI-1-luc1). In mAPSCs, FOXC1 overexpression was able to significantly activate luciferase expression by way of the reporter pSTI-1-luc1 containing the FOXC1-responsive element (FRE) (Figure 3D). In contrast, a reporter without the FRE (pSTI-1-luc2) and with a mutation of the putative FOXC1-binding site (pSTI-1-mutFRE) completely abolished the FOXC1 responsiveness of the construct, demonstrating that the 5'-**AAGCAAATA**-3' site mediated the FOXC1 response (Figure 3D). These results indicate that the STI-1 promoter contains at least one functional FRE. ChIP assays using mAPSCs further demonstrated FOXC1 binding to the endogenous promoter region of STI-1 containing the FRE (Figure 3E). Taken together, these results suggest that FOXC1 transcription factors induce the transcription of STI-1 in mAPSCs.

### FOXC1-induced STI-1/PrP^C^ autocrine signaling regulates mAPSCs self-renewal and proliferation

**Figure 4:**
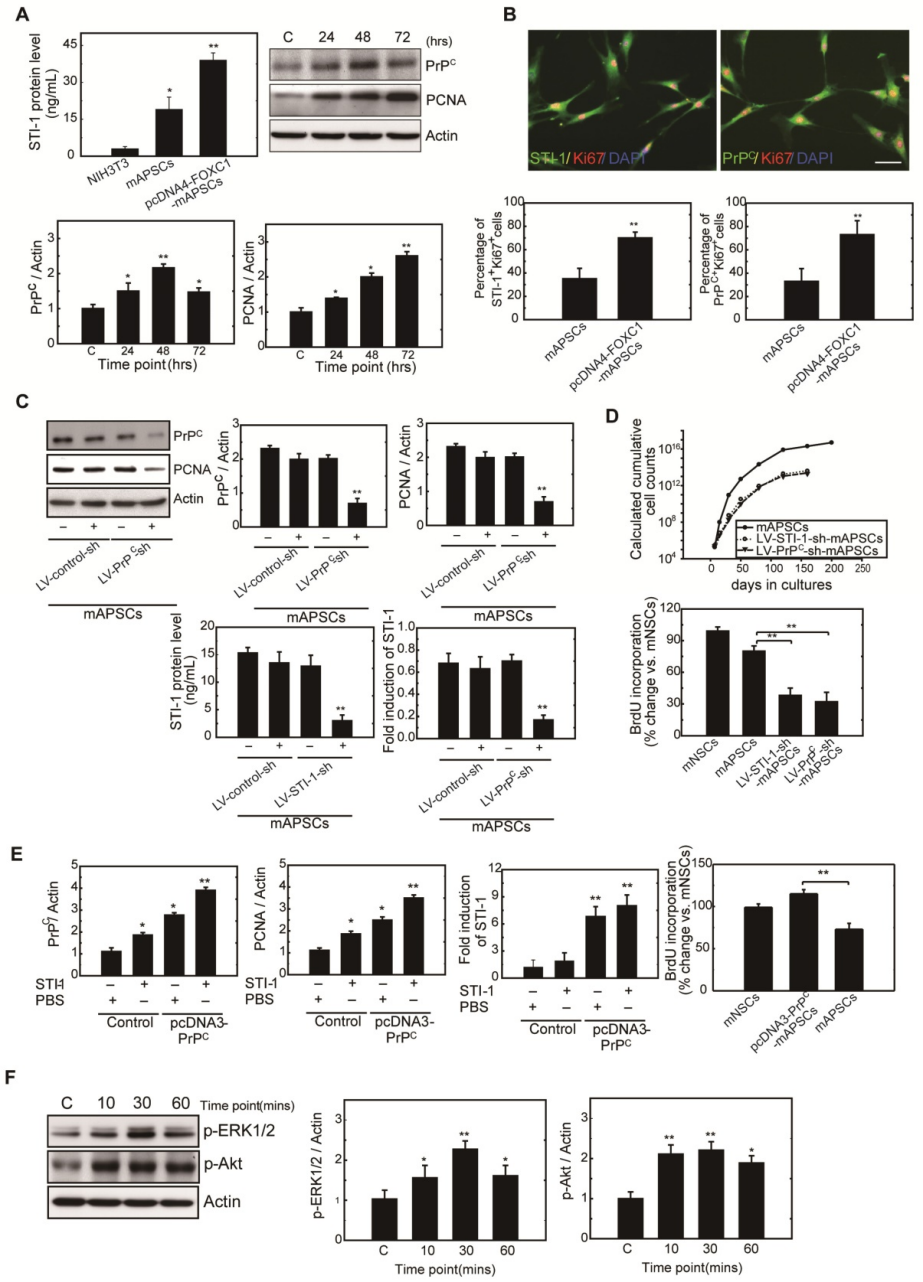
** mAPSC self-renewal and proliferation were regulated by FOXC1-mediated STI-1/PrP^C^ autocrine signaling.** (**A**) Significantly increased levels of STI-1 in culture medium by ELISA were found in pcDNA4-FOXC1-mAPSCs compared to the control (NIH3T3 cells as a negative control). Additionally, a significant increase in expression of PrP^C^ and PCNA by western blot was observed in pcDNA4-FOXC1-mAPSCs relative to the control. (**B**) A greater number of STI-1^+^Ki67^+^ and PrP^C+^Ki67^+^ mAPSCs by immunofluorescence analyses was present in pcDNA4-FOXC1-mAPSCs than in the control. (**C**) Significant downregulation of PrP^C^ and PCNA was found in LV-PrP^C^-sh-mAPSCs compared with the control-shRNA. In ELISA, a reduction of STI-1 expression was revealed both in LV-STI-1-sh-mAPSCs and LV-PrP^C^-sh-mAPSCs. (**D**) LV-STI-1-sh-mAPSCs and LV-PrP^C^-sh-mAPSCs showed a reduction of growth kinetics and BrdU incorporation. (**E**) After STI-1 treatment for 48 hr, a significantly increased PrP^C^ and PCNA expression was found in PrP^C^-overexpressed mAPSCs compared with the control. Sustained elevation level of STI-1 by ELISA was observed in pcDNA3.1-PrP^C^-mAPSCs following STI-1 treatment. In addition, pcDNA3.1-PrP^C^-SCs revealed an increase of BrdU incorporation. (**F**) In the signaling cascade, a time-dependent activation of p-ERK1/2 and p-Akt pathways was found following recombinant STI-1 treatment of mAPSCs. Data are expressed as the mean ± SEM. * *P* < 0.05 and ** *P* < 0.01 vs. control, Bar = 40 μm

To determine whether FOXC1 regulates mAPSC proliferation through STI-1/PrP^C^ signaling, ELISA, western blot and immunofluorescence studies were performed with mAPSCs transfected with pcDNA4-FOXC1 (pcDNA4-FOXC1-mAPSCs). In the ELISA study, FOXC1-overexpressed mAPSCs (per 10^6^ cells) showed significantly increased levels of STI-1 in culture medium compared to mAPSCs without FOXC1 transfection and in NIH3T3 fibroblasts (NIH3T3 cells as a negative control) (Figure 4A). Western blots revealed a significant increase in expression of PrP^C^ and markers of proliferative cell nuclear antigen (PCNA) in FOXC1-overexpressed mAPSCs relative to the control (Figure 4A). Moreover, immunofluorescence analyses demonstrated that greater levels of STI-1^+^Ki67^+^ and PrP^C+^Ki67^+^ mAPSCs were found in the pcDNA4-FOXC1-mAPSCs than in controls (Figure 4B). We therefore speculate that STI-1 secreted from mAPSCs may act via PrP^C^ on these cells in an autocrine fashion, thus contributing to their proliferation.

Next, examining FOXC1-modulated mAPSC proliferation via STI-1/PrP^C^ signaling using shRNA techniques against PrP^C^, immunoblotting revealed that protein expression levels of PrP^C^ and PCNA were significantly inhibited compared with the control-shRNA (Figure 4C). Consistently, downregulation of STI-1 expression measured by ELISA was noted both in LV-STI-1-sh-mAPSCs and LV-PrP^C^-sh-mAPSCs (Figure 4C). In addition, mouse neural stem cells (mNSCs) serving as a positive control of highly proliferating cells, an increase of growth kinetics and BrdU incorporation were abolished in LV-STI-1-sh-mAPSCs and LV-PrP^C^-sh-mAPSCs (Figure 4D). These data suggest that STI-1/PrP^C^ autocrine signaling is required for maintaining mAPSC proliferation.

To determine whether triggering STI-1/PrP^C^ signaling induces mAPSC self-renewal, PrP^C^ cDNA was transfected into mAPSCs (pcDNA3-PrP^C^-mAPSCs). We observed that PrP^C^ and PCNA expression were increased in these PrP^C^-overexpressed mAPSCs when exposed to STI-1 (100 ng/mL) for 48hr compared with the control (Figure 4E). The ligand-induced activation of PrP^C^ could also induce and continuously maintain elevated levels of STI-1 expression as determined by ELISA in pcDNA3-PrP^C^-mAPSCs (Figure 4E). Moreover, an increase of BrdU incorporation was found in pcDNA3-PrP^C^-mAPSCs (Figure 4E). Taken together, these findings suggest that the STI-1 autocrine signaling pathway also operates in a self-stimulating fashion and contributes to proliferation and self-renewal in mAPSCs.

To determine whether STI-1 can activate the downstream MAPK cascade, mAPSCs that endogenously express PrP^C^ were treated with recombinant STI-1 protein. STI-1 (100 ng/mL, R&D Systems) treatment increased the expression of p-ERK1/2 and p-Akt in mAPSCs in a time-dependent manner (Figure 4F), which was inhibited by specific inhibitors of PD98059 or LY294002 (data not shown). These results suggest that ERK1/2 and Akt are key elements in the FOXC1-STI-1-PrP^C^ signaling pathway.

### STI-1 is an essential factor in mAPSCs for NPC self-renewal

**Figure 5:**
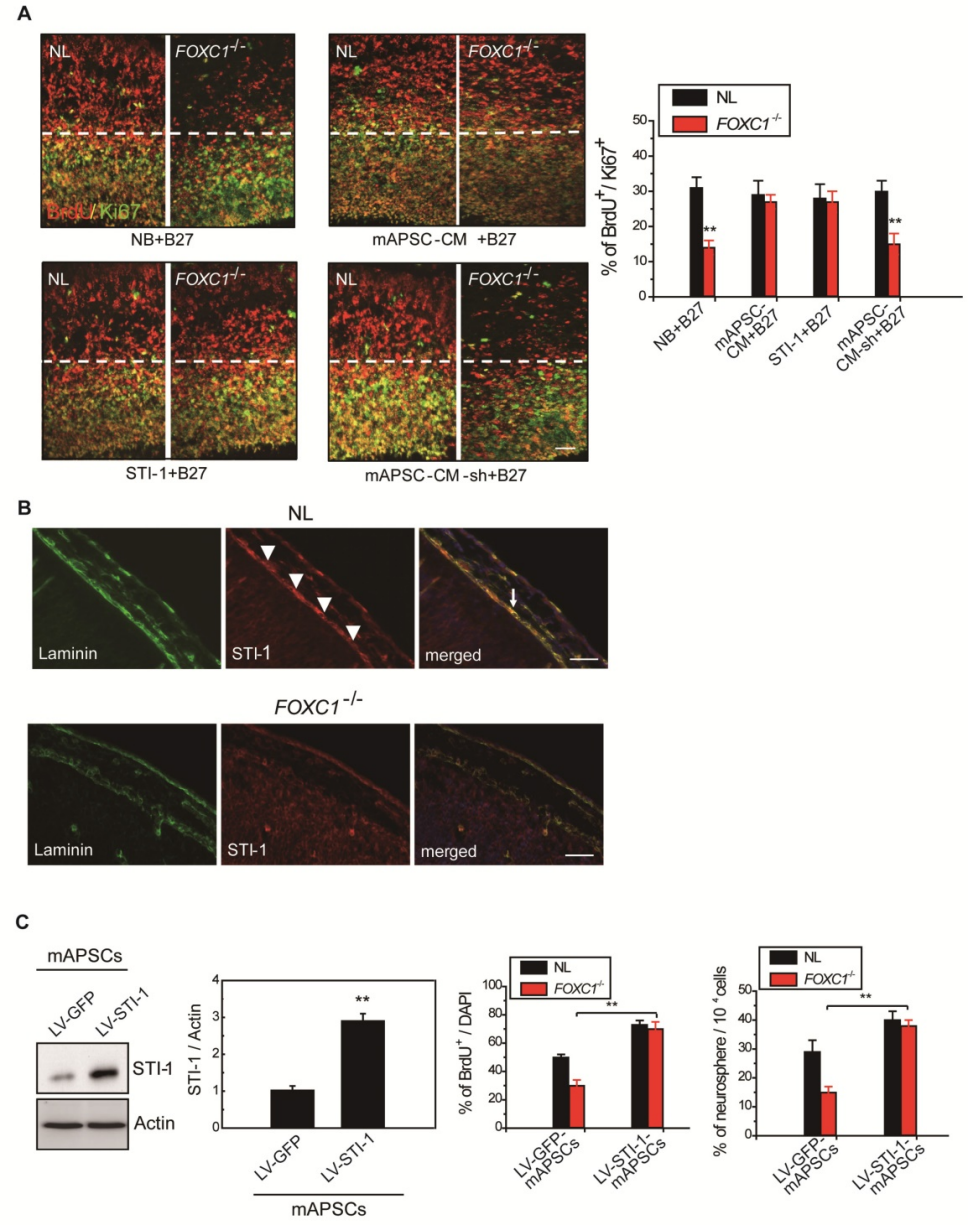
** Meningeal-derived STI-1 rescues deficient cortical neurogenesis caused by lack of FOXC1.** (**A**) Representative images of BrdU^+^/Ki67^+^ double immunostaining; the dotted line demarcates the transition from proliferative and postmitotic zones in the ventricular wall. In co-culture experiments, a significantly enhanced proportion of BrdU^+^/Ki67^+^ cells were found in *FOXC1^-/-^* forebrain explants embedded in mAPSCs-CM, but not in that of NLs. The cortex of *FOXC1^-/-^*brain explants treated with STI-1 (100 ng/mL) exhibited increased cell cycle exit; no increase was observed in brain explants of NLs. However, rescue of the cell-cycle exit phenotype in the *FOXC1^-/-^* brain explant was inhibited in the NL-mAPSC-CM-sh. (**B**) In an immunohistochemistry study (E14.5), a higher STI-1 expression that colocalized with laminin was observed in the meninges of NLs compared to *FOXC1^-/-^* mice meninges. STI-1 signal was co-expressed with LacZ staining in the *FOXC1^+/-^* heterozygous mice meninges. (**C**) Significant overexpression of STI-1 was found in LV-STI-1 transduction in NL-mAPSCs (LV-STI-1-NL-mAPSCs) compared with the control (LV-GFP-NL-mAPSCs). Reversion of the *FOXC1^-/-^* SVZ neurosphere phenotype after co-culture with LV-STI-1-NL-mAPSCs was demonstrated by increased numbers of neurosphere formation and percentage of BrdU^+^ cells. Data are expressed as the mean ± SEM. * *P* < 0.05 and ** *P* < 0.01 vs. control, Bar = 40 μm

Gradual reduction in NPC production when meningeal coverage declines suggests that cortical neurogenesis in *FOXC1^-/-^*mice represents a graded loss in a meningeal signal. To test this hypothesis, we performed co-culture of *FOXC1^-/-^* explants (E13.5 forebrain explants) with mAPSCs that conditioned the shared media (mAPSC-CM). mAPSCs co-cultured with *FOXC1^-/-^* forebrain explants significantly increased the proportion of BrdU^+^/Ki67^+^ cells but did not affect cell-cycle exit in the NL forebrain explants (Figure 5A). In addition, treatment with STI-1 (100 ng/mL) and B27 supplement increased cell cycle exit in the cortex of *FOXC1^-/-^*brain explants (Figure 5A) but not in that of NLs. We next determined whether STI-1 is a required component of mAPSC-CM for rescue of *FOXC1^-/-^* slice explants. LV-STI-1-sh-infected mAPSCs (LV-STI-1-sh-mAPSCs) were co-cultured with slices in media containing B27 supplement, and STI-1 levels were assayed by ELISA (USCN) (Figure 5A). Although conditioned media's lack of STI-1 in LV-STI-1-sh-mAPSCs (mAPSC-CM-sh) did not alter cell-cycle exit in NL slices, the conditioned media failed to rescue the cell-cycle exit phenotype in the *FOXC1^-/-^* slices (Figure 5A).

We next examined the expression of STI-1 in meninges of NL and *FOXC1^-/-^* mice. In E13.5 mice, NL tissue showed higher expression of STI-1 in the meninges by immunohistochemistry compared to that of *FOXC1^-/-^* mice (Figure 5B). However, STI-1 expression in the *FOXC1^-/-^* mouse skull was reduced and patchy in the dorsal meningeal areas (Figure 5B).

### STI-1 expression compensates for FOXC1^-/-^ embryonic neocortical neurosphere self-renewal and proliferation

We hypothesized that if STI-1 compensates for the embryonic neocortical cells proliferation under FOXC1 inactivation, co-culture of STI-1-overexpressed mAPSCs produced by LV-STI-1 infection (LV-STI-1-mAPSCs) with *FOXC1^-/-^*embryonic neocortical neurospheres would enhance the proliferative potential of neurospheres. To gain further insight into the molecular mechanisms underlying the involvement of FOXC1 and STI-1 in embryonic neocortical neurosphere proliferation, we overexpressed STI-1 in mAPSCs to determine whether increased STI-1 secretion from mAPSCs would enhance cell proliferation and renewal of co-cultured neurospheres to overcome the loss of FOXC1. Transduction of LV-STI-1 greatly upregulated STI-1 in mAPSCs compared with the control (LV-GFP-mAPSCs) (Figure 5C). Co-culture of LV-STI-1-mAPSCs reversed the phenotype in *FOXC1^-/-^* embryonic neocortical neurospheres as demonstrated by increasing numbers of neurosphere formation (Figure 5C) and cell proliferation assays that evaluated the percentage of BrdU^+^ cells (Figure 5C). In both sets of experiments, the behavior of *FOXC1^-/-^* neurospheres co-cultured with mAPSCs was indistinguishable from their NL counterparts, which indicates that restoring STI-1 levels from mAPSCs in *FOXC1^-/-^* embryonic neocortical cells is sufficient to compensate for FOXC1 loss.

### Administration of APSCs modulated neurite regeneration in vivo and in vitro

**Figure 6:**
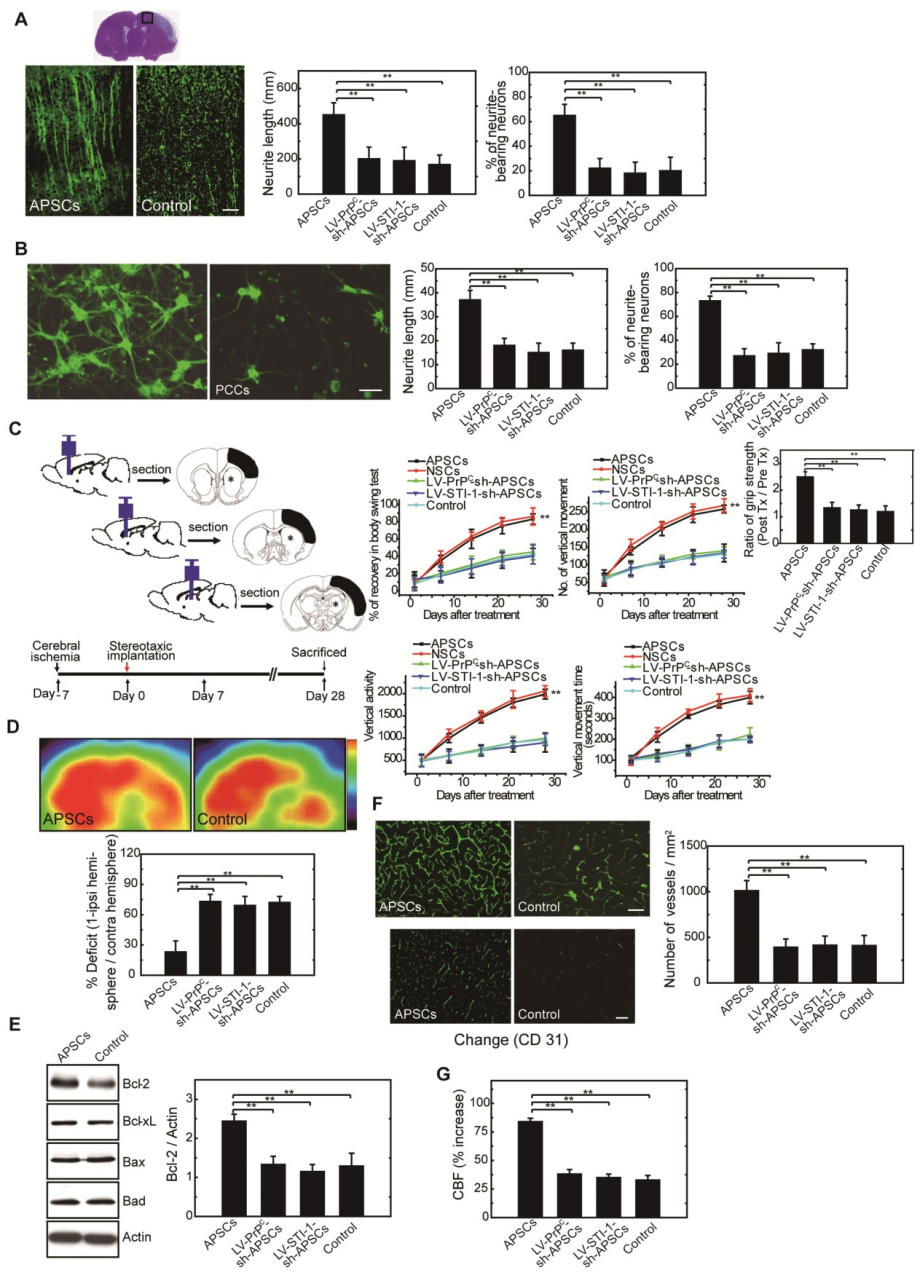
** APSCs induced neural regeneration in vitro and in vivo.** (**A**) By measuring the length of neurites and the number of neurite-bearing neurons, rats receiving APSC transplantation showed significantly increased neural regeneration in the penumbral area of the rat brain at 28 days after cerebral ischemia compared to control rats, but not in rats transplanted with LV-PrP^C^-sh-APSCs or LV-STI-1-sh-APSCs. (**B**) In an OGD model, APSC co-culture significantly enhanced the neurite length and the number of neurite-bearing neurons of PCCs compared to controls, but not in PCCs co-cultured with LV-PrP^C^-sh-APSCs or LV-STI-1-sh-APSCs. (**C**) A significant recovery of one-month long term neurological tests including body symmetry, locomotor activity (vertical activity, vertical movement time, and the number of vertical movements), and grip strength were found in stroke rats transplanted with APSCs compared to controls. However, rats treated with LV-PrP^C^-sh-APSCs or LV-STI-1-sh-APSCs exhibited no neurological improvements. (**D**) In a functional ^18^FDG-PET study, a significant increase in glucose metabolism was noted in stroke rats with APSC implantation compared to controls but not in rats treated with LV-PrP^C^-sh-APSCs or LV-STI-1-sh-APSCs. (**E**) At 3 days after implantation, a significant upregulation of Bcl-2 was observed in the APSC-treated stroke rats compared to controls. Consistently, LV-PrP^C^-sh-APSC or LV-STI-1-sh-APSC implantation abolished the increased expression of Bcl-2. (**F**) Stereotaxic implantation of APSCs enhanced the FITC-dextran-perfused microvasculature and increased the number of CD31^+^ neovasculature in the stroke brain. This phenomenon was not found in LV-PrP^C^-sh-APSC- and LV-STI-1-sh-APSC-treated animals. (**G**) Increased functional cerebral blood flow (CBF) by laser doppler flowmetry (LDF) was observed in APSC-implanted rats but not in rats implanted with LV-PrP^C^-sh-APSCs and LV-STI-1-sh-APSCs. Data are expressed as the mean ± SEM. * *P* < 0.05 and ** *P* < 0.01 vs. control, Bar = 40 μm

To investigate neurite regeneration after intracerebral APSC transplantation, we measured the length of neurites and the number of neurite-bearing neurons in the penumbral area of the ischemic rat brain at 28 days after cerebral ischemia. The length of neurites was significantly longer in rats that received the APSC-transplantation compared to control rats (Figure 6A). Likewise, the number of neurite-bearing neurons was significantly increased in rats that received APSC-transplantation compared to control rats (Figure 6A). Consistent with the essential role of PrP^C^ and STI-1 in this regard, transplantation of LV-PrP^C^-sh-treated APSCs (LV-PrP^C^-sh-APSCs) or LV-STI-1-sh-treated APSCs (LV-STI-1-sh-APSCs) completely abolished the increase in neurite length and the increase in the number of neurite-bearing neurons compared to that of APSCs in stroke rats. (Figure 6A).

To evaluate APSC-mediated neurite regeneration following oxygen-glucose deprivation (OGD) in vitro, PCCs were co-cultured with APSCs (Figure 6B). Co-culturing with APSCs significantly increased the average neurite length and the number of neurite-bearing neurons of PCCs following OGD. Consistent with the requirement for PrP^C^ and STI-1 in this regard, the beneficial effect of APSC co-culturing on PCCs was completely abolished by infection with LV-PrP^C^-sh or LV-STI-sh (Figure 6B).

### Intracerebral APSC transplantation improved neurological behavior after cerebral ischemia

One-month long-term neurological tests were performed in rats subjected to focal cerebral ischemia after implanting each rat with APSCs or vehicle control. Rats implanted with APSCs had improved body symmetry, locomotor activity (vertical activity, vertical movement time, and number of vertical movements), and grip strength (Figure 6C) compared to control rats (vehicle-treated) with no stem cell implantation. In marked contrast, these beneficial effects of APSC implantation, including improvements in body symmetry, locomotor activity, and grip strength, were completely reversed by injecting rats with LV-PrP^C^-sh-APSCs or LV-STI-sh-APSCs (Figure 6C).

### Glucose metabolic activity was enhanced in APSC-treated stroke rats

Each rat was examined by ^18^FDG-PET scanning to determine whether intracerebral APSCs improved glucose metabolism following cerebral ischemia. MicroPET images showed an ~60% deficit in glucose metabolism in the ischemic right cortex of control animals relative to their nonstroked left cortex. Implantation of APSCs into the ischemic cortex significantly recovered glucose metabolism compared to control animals with no implantation (Figure 6D). Moreover, APSC implantation-mediated recovery was completed inhibited by APSCs infected with LV-PrP^C^-sh or LV-STI-sh (Figure 6D).

### Upregulation of anti-apoptotic protein in APSC-treated stroke rats*.*

In investigations of the mechanism by which APSC implantation improved stroke outcome, the expression of apoptosis-related proteins was examined 3 days after implantation of APSCs or vehicle. The expression of anti-apoptotic protein Bcl-2 in rats subjected to cerebral ischemia was markedly increased by implantation of APSCs. This suggested that engraftment of APSCs can induce neuroprotection by upregulating anti-apoptotic proteins in the ischemic brain. Additionally, the increase in Bcl-2 was completed inhibited by treating rats with LV-PrP^C^-sh- or LV-STI-sh-APSCs (Figure 6E).

### Intracerebral APSC transplantation induced angiogenesis to facilitate cerebral blood flow (CBF)

To determine whether APSCs could induce angiogenesis, double immunofluorescence staining, FITC-dextran perfusion studies, and blood vessel density assays were performed on brain slices from APSC-treated or vehicle-control-treated rats. Clearly, APSC implantation significantly enhanced cerebral microvascular perfusion with FITC-dextran compared to controls (Figure 6F).

Moreover, ischemic rats treated with APSCs showed more CD31^+^ neovasculature in the penumbric region compared to control rats (Figure 6F). However, this increase in the number of vessels (CD31^+^) was abolished by implantation of LV-PrP^C^-sh-APSCs and LV-STI-sh-APSCs in stroke rats (Figure 6F).

To assess whether this increased vascular density functionally increased cerebral blood flow (CBF) into the ischemic brain, experimental rats were monitored by laser doppler flowmetry (LDF) under anesthesia one week after cerebral ischemia. As expected from the increased vascular network, rats receiving APSC implantations exhibited increased regional CBF in the ischemic cortex compared to vehicle controls, and this increase in CBF was inhibited by injection of LV-PrP^C^-sh-APSCs and LV-STI-sh-APSCs in stroke rats (Figure 6G).

## Discussion

In vertebrate cranial development, cranial neural crest cells (NCC) spread and ultimately differentiate into the bones of the face and the frontal bone of the skull, pericytes and blood vessel smooth muscle cells (cephalic mural cells) as well as the meninges 3, 10. The meninges, during embryo development, form two different layers: the pachymeninges and the leptomeninges. The pachymeninges (dura mater) is an external and thick layer of mesenchymal composition, while the leptomeninges (arachnoid and pia mater) is a double and thinner layer comprising both mesenchymal and neural crest cells. The dural and leptomeninges surrounding the forebrain are neural crest derived, the dural and leptomeninges surrounding the remainder of the brain and spinal cord are mesoderm derived 3. The highly migratory and plastic neural crest cells ultimately develop into many diverse tissues in the body including peripheral nerves, melanocytes, bone, cartilage, muscle, heart, and adrenal glands 56, 57. Indeed, evidence of neural crest origins of the arachnoid-pia tissue stimulated our search for multipotential stem cells residing in the arachnoid-pia tissue that are capable of differentiating into neuroglial cells and other cells of mesenchymal origin. Recent studies have found that nestin^+^ leptomeningeal-derived neural progenitor cells (NPCs) were identified from this nonconventional neurogenic region of adult leptomeningeal cells following cortical infarction and spinal cord injury 5, 7-9. These leptomeningeal-derived NPCs also exhibit self-renewal and proliferation capabilities and possess multipotent differentiation potential 5, 7. Furthermore, nestin^+^NG2^+^ pericytes located near the perivascular region of the leptomeninges might be the potential source of these leptomeningeal-derived NPCs 5, 8. Therefore, in this study, we isolated and characterized the NGFRp75^+^ arachnoid-pia stem cells (APSCs) from adult human and mouse arachnoid-pia tissue (mAPSCs). Additionally, APSCs express a neural crest cell lineage marker (Wnt1), ES cell markers (OCT4, SOX2, SSEA4 and SSEA3), neural progenitor markers (nestin, musashi-1 and NGFRp75) and mesenchymal stem cell markers, and they also possess self-renewal and multipotent differentiation potential. APSCs were able to form spheres in culture and differentiate into neuronal, glial, oligodendrocyte, adipocyte, osteocyte, chondrocyte and endothelial cells. Because specific markers of microvascular pericytes are nestin and NG2 58, the nestin^+^NGFRp75^+^ APSCs might develop from perivascular NGFRp75-expressing pericytes 59. Regarding the relationship between FOXC1 expression and meninges, diminished FOXC1 protein expression in all three layers of meningeal cells was observed in FOXC1 mutant mice and contributes to the cortical and skull defects 18. Abnormal differentiation of arachnoid-pia cells was found in the meninges of FOXC1 mutant mice 17, 18. Based on the evidence of FOXC1 mutant mice with defects in forebrain meningeal formation 17, it is reasonable to speculate that a highly proliferative stem cell derived from arachnoid-pia tissue might be regulated by FOXC1. Consistent with our hypothesis of a potential source of APSCs from microvascular pericytes, FOXC1 expression also regulates pericyte biology 18, 44. Thus, in this study, we demonstrated that downregulation of FOXC1 in mutant mice abolished the self-renewal potential, indicating that FOXC1 regulates APSC proliferation. In addition, lack of FOXC1 disrupts cell cycle progression, causing an increase in G0/G1 cells and a concomitant decrease in S and G2/M cells. In summary, diminished expression of FOXC1 might disturb the self-renewal and proliferative potential of APSCs and result in abnormal development of cortical and skull formation.

While cortical neurogenesis proceeds, radial expansion of the cortex begins as radial glial cells in the ventricular zone (VZ) differentiate asymmetrically to generate mature neurons directly targeting additional neuronal divisions 60, 61. Several intracellular molecules, such as transcription factors or extracellular substances such as growth factors, have been implicated in initiating this important process 28, 62. However, it remains largely unknown as to which factors control the molecular mechanisms of cortical neurogenesis. Previous studies have shown that the inner layer of meningeal cells secreting the stromal cell-derived factor 1α (SDF-1α) exert a potent chemoattractive influence over migrating Cajal-Retzius (CR) cells, which are critical in the development of the cerebral cortex 63, 64. The dura, which is the outer layer of the meninges, releases fibroblast growth factor-1 (FGF-2) to induce skull bone formation 65. Moreover, FOXC1 mutant mice with defects in forebrain meningeal formation due to a loss of meningeal-secreted retinoic acid (RA) exhibit a dramatic reduction in both neuron and progenitor cell production 1. In this study, we first identified the unique mAPSCs derived from meninges that are regulated by the expression of FOXC1. Importantly, STI-1 that is transcriptionally regulated by FOXC1 was found to be an important growth factor for stimulating the proliferation and self-renewal of APSCs. Regarding the FOXC1 binding site in the regulated gene promoter, such as CXCR4, SDF-1α, and TGF-β2 66-68, we identified a FOXC1 responsive-element (FRE) of 5'-**AAGCAAATA**-3' in the STI-1 promoter region (-180 to -172) based on the rule of the consensus-binding sequence 66, 69. We proved this hypothesis using ChIP analysis. In addition, we verified that forced overexpression of FOXC1 in transfected mAPSCs leads to a significant upregulation of reporter activity in the luciferase construct containing FRE. Moreover, STI-1/PrP^C^ autocrine signaling loops were also observed to regulate the proliferation and self-renewal of mAPSCs. Therefore, we provide novel insights into newly identified APSCs secreting STI-1, which might represent another signaling pathway for neurogenesis. Regarding the status of STI-1 in the mAPSCs proliferation, as we knew that retinoic acid (RA) from meninges play a significant role in the cortical neurogenesis. Based on our current results, STI-1 might be one of the other factors contributing on the stem cells self-renewal during developmental stage. We speculate that both RA and STI-1 would provide a synergistic contribution over the neurogenesis. Further investigations need to disclose these molecular mechanisms.

Regarding the autocrine action, soluble STI-1 binding to PrP^C^ exerts autocrine/paracrine activity, which stimulates neuritogenesis, neuroprotection, and memory formation 21, 23, 24, 70. STI-1 is also a trophic factor for glial cells that modulates astrocyte proliferation as an autocrine through PKC activation 71. Furthermore, Hop (Hsp70/Hsp90 organizing protein), also known as STI-1, facilitates the phosphorylation activator in embryonic stem cells (ESCs) and thus implies an important role in pluripotency signaling 72. Consistently, both of the chaperones Hsp70 and Hsp90, the main partners of STI-1, have been definitively associated with survival and proliferation of neural stem/progenitor cells (NPCs) 73, 74. Recently, STI-1 was shown to be secreted by neurospheres, which is consistent with its characterization above as an autocrine neurotrophic factor 20. In this study, we isolated unique meninges-derived APSCs, which harbor ESC markers (OCT4 and SOX2), MSCs (CD44, CD90, etc.) and NPCs (nestin, musashi-1 and NGFRp75). We also provide evidence that loss of FOXC1 might disturb the self-renewal and proliferative potential of APSCs. As a FOXC1-targed gene, STI-1 exerts downstream modulation of survival and proliferation of APSCs through an autocrine signaling pathway.

## Supplementary Material

Supplementary figures.

## Figures and Tables

**Table 1:** Quantitative RT-PCR primers list

Gene	Sequence	Amplicon (bps)
**OCT4**	**F:** 5'-GCCAAGCTGCTGAAACAGAAG-3' **R:**5'-CTGGCTGAACACCTTTCCAAA-3'	96 bps
**SOX2**	**F:** 5**'-**CGCCGAGTGGAAACTTTTGT-3' **R:**5'-CGCGGCCGGTATTTATAATC-3'	111 bps
**Nanog**	**F:** 5'-GGCCTGACTCAGAAGGGCTC-3' **R:**5'-TGCCCCATACTGGAAGGTTTC-3'	106 bps
**Nestin**	**F:** 5'-GCAACTGGCACACCTCAAGA-3' **R:**5'-GGGTCCAGAAAGCCAAGAGAA-3'	129 bps
**NGFRp75**	**F:** 5'-CCTACGGCTACTACCAGGATGAG-3' **R:**5'-TGGCCTCGTCGGAATACG-3'	147 bps
**Musashi-1**	**F:** 5'-GTTTCGGCTTCGTCACTTTC-3' **R:**5'-GAGTCACCATCTTGGGCTGT-3'	112 bps
**Fabp7**	**F:** 5'-TCGGTTGGATGGAGACAAGC-3' **R:**5'-TCCCCAAAGGTGAGAGTCACA-3'	110 bps
**Smad4**	**F:** 5'-GCACTACCACCTGGACTGGAA-3' **R:**5'-TGTGAACCGGCCAGTAATGTC-3'	126 bps
**Pax6**	**F:** 5'-CAACCTGGCTAGCGAAAAGC-3' **R:**5'-CGTCTTGCGTGGGTTGC-3'	145 bps
**Dcx**	**F:** 5'-AAAGCTTCCCCAACACCTCA-3' **R:**5'-CCATTTGCGTCTTGGTCGTTA-3'	101 bps
**Olig2**	**F:** 5'-CTGGCGCGAAACTACATCCT-3' **R:**5'-GTGGTGACCCCCGTAAATCTC-3'	84 bps
**Galc**	**F:** 5'-ACTTCGGTGCCTCTCTGCAT-3' **R:**5'-AGGGTTCAGTGCCGTCTGTT-3'	75 bps
**SDF-1α**	**F:** 5'-ATCAGTGACGGTAAGCCAGTCA-3' **R:**5'-TGCTTTTCAGCCTTGCAACA-3'	145 bps
**CXCR4**	**F:** 5'-CGAGCATTGCCATGGAAATAT-3' **R:**5'-ATTGCCCACTATGCCAGTCAA-3'	170 bps
**STI-1**	**F:** 5'-ACTCTCAGCGTCCTCCTTGG-3' **R:**5'-GGTGGAGGTGTTGCAATCTCTT-3'	71 bps
**PrP^C^**	**F:** 5'-GTCAATTCTTCAACGTCGGTGTG-3' **R:**5'-TCCCATAGCAAACACATCTACCG-3'	79 bps

## Abbreviations

APSCs: human arachnoid-pia stem cells
mAPSCs: mouse arachnoid-pia stem cells
NCSCs: neural crest stem cells
NSCs: neural stem cells
FOXC1: forkhead box family C1
STI-1: stress-inducible protein 1
PrP^C^: cellular prion protein
OGD: oxygen-glucose deprivation
FDG-PET: [^18^F]fluoro-2-deoxyglucose positron emission tomography
NPCs: neural progenitor cells
GFAP: glial fibrillary acidic protein
MAP-2: microtubular associated protein-2
Neu-N: neuronal nuclear
