# Supplementary material for: Role of FOXC1 in regulating APSCs self-renewal via STI-1/PrPC signaling
Source: Theranostics. 2019 Aug 15;9(22):6443–65. doi: 10.7150/thno.35619 (PMC6771253; doi:10.7150/thno.35619)
Supplement: Supplementary file 1 — Supplementary figures. [file thnov09p6443s1.pdf]

SUPPLEMENTARY METERIALS

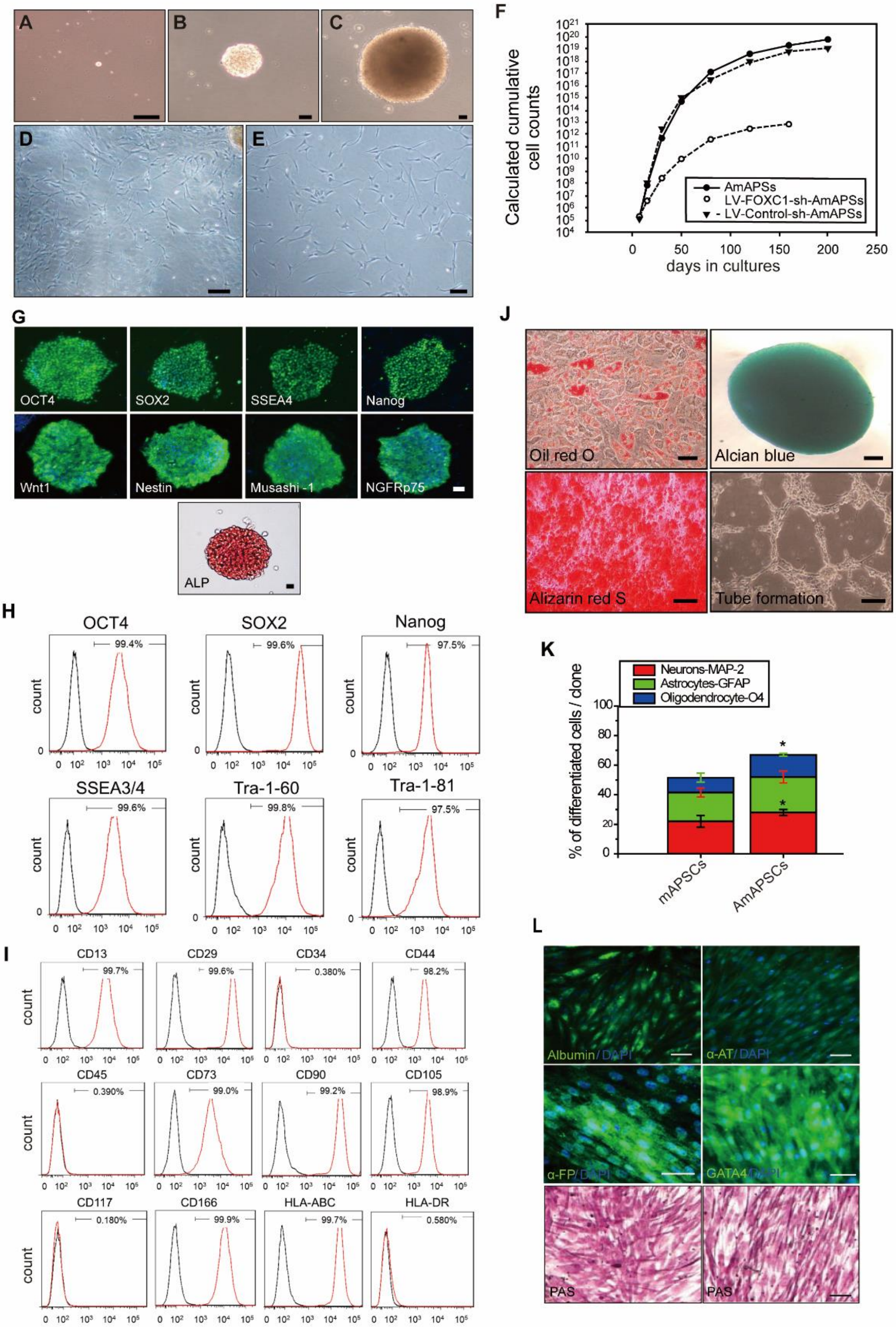

Figure S1. Isolation and characterization of arachnoid-pia stem cells (AmAPSCs) from adult mouse meninges. (**A**) Representative images of AmPSCs at 5-7 days; some cell clusters started to form in the primary culture of arachnoid-pia tissues from

adult human meninges. (**B-C**) After 10-14 days, 3-dimensional (3D) arachnoid-pia spheres (mAPSs) adhered to plastic wells, and only a small number of spheres floated. (**D-E**) Many neuroglia-like cells migrated from the spheres and show a unique morphology. (**F**) The growth kinetic analysis data showed a reduction of proliferative cell numbers in the LV-FOXC1-sh-AmAPSs. (**G**) In immunocytochemistry (IHC), a neural crest cell marker (Wnt1), four ES cell markers (OCT4, SOX2, SSEA4 and Nanog) and positive ALP staining were consistently expressed in most of the mAPSs. Representative NSC markers of nestin, musashi-1 and NGFRp75 were also strongly immunoreactive. (**H-I**) Flow cytometric analysis in isolated cells from AmAPSs was positive for ES cell markers and mesenchymal stem cell origin but negative for hematopoietic stem cell origin. (**J**) In *in vitro* differentiation assays, AmAPSCs differentiated into mesenchymal phenotypes (adipocyte: oil-red-O staining; chondrocyte: alcian blue staining; and osteocyte: alizarin red staining in the lower panel) (**K**) The expression of MAP-2, GFAP and O4 were higher in AmAPSCs than mAPSCs (**L**) In endodermal differentiation, immunocytochemical analysis of differentiated hepatocytes was positive for human albumin, α-feto-protein (α-FP), α1-antitrypsin (α1-AT) and GATA4. Glycogen storage in the hepatocyte was examined by PAS staining. Data are expressed as the mean ± SEM. * $P < 0.05$ and * $P < 0.01$ vs. control, Bar = 40 μm

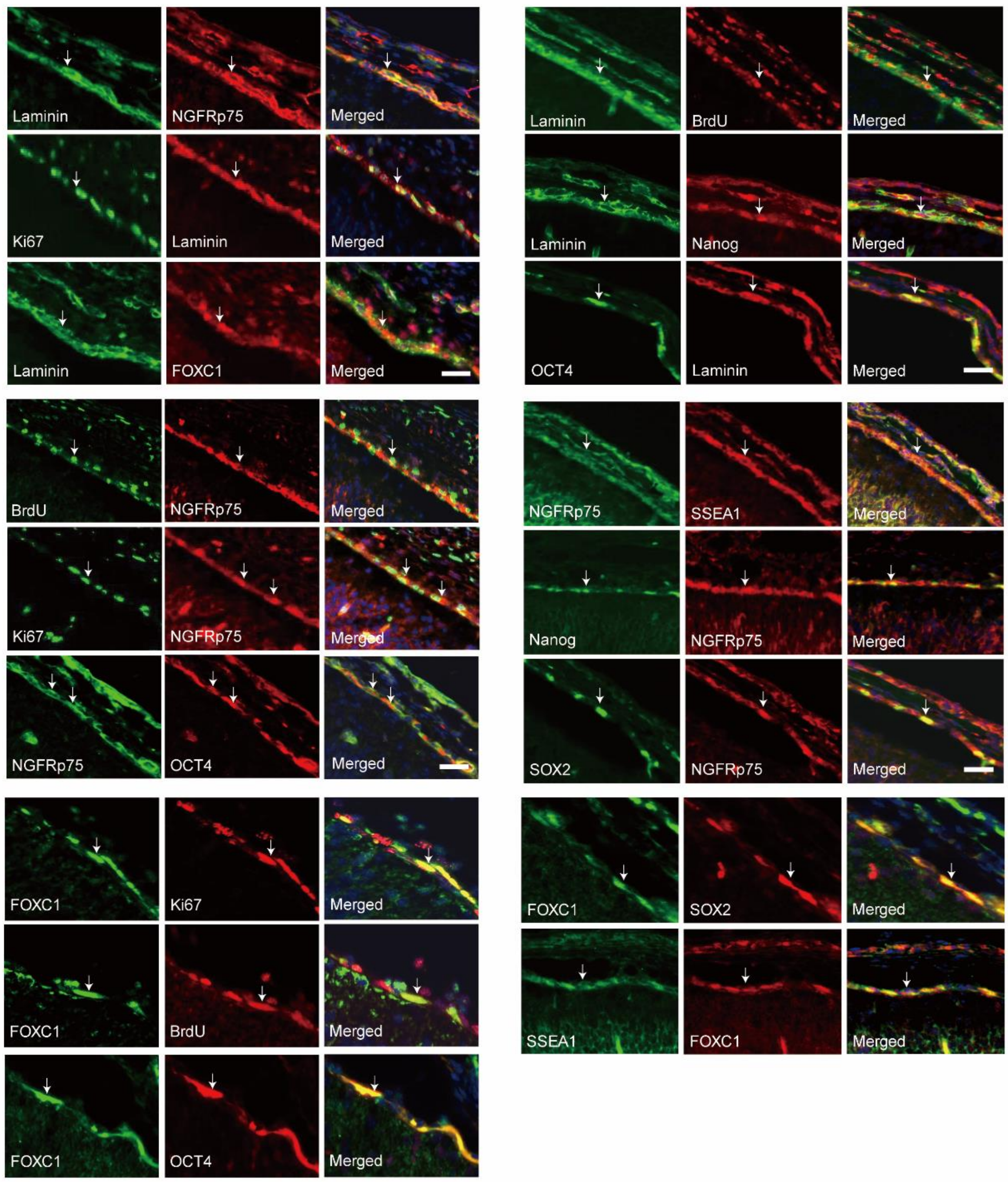

Figure S2. Double immunofluorescence study of laminin$^+$NGFRp75$^+$ and laminin$^+$FOXC1$^+$ cells. laminin and NGFRp75 immunopositive cells were co-expressed with proliferative markers (BrdU and Ki67) and ES cell markers (OCT4, SOX2, SSEA-1 and Nanog).
